# Multi-Electrode Array Analysis Identifies Complex Dopamine Responses and Glucose Sensing Properties of Substantia Nigra Neurons in Mouse Brain Slices

**DOI:** 10.3389/fnsyn.2021.635050

**Published:** 2021-02-26

**Authors:** Nadja Mannal, Katharina Kleiner, Michael Fauler, Antonios Dougalis, Christina Poetschke, Birgit Liss

**Affiliations:** ^1^Institute of Applied Physiology, University of Ulm, Ulm, Germany; ^2^Linacre and New College, University of Oxford, Oxford, United Kingdom

**Keywords:** glucose-excited GE-neurons, dopamine excited neurons, dopamine inhibited neurons, dopamine-autoreceptor, neuronal calcium sensor NCS-1, GIRK channel, dopamine receptor desensitization, glucose-responsive neurons

## Abstract

Dopaminergic (DA) midbrain neurons within the substantia nigra (SN) display an autonomous pacemaker activity that is crucial for dopamine release and voluntary movement control. Their progressive degeneration is a hallmark of Parkinson's disease. Their metabolically demanding activity-mode affects Ca^2+^ homeostasis, elevates metabolic stress, and renders SN DA neurons particularly vulnerable to degenerative stressors. Accordingly, their activity is regulated by complex mechanisms, notably by dopamine itself, via inhibitory D2-autoreceptors and the neuroprotective neuronal Ca^2+^ sensor NCS-1. Analyzing regulation of SN DA neuron activity-pattern is complicated by their high vulnerability. We studied this activity and its control by dopamine, NCS-1, and glucose with extracellular multi-electrode array (MEA) recordings from midbrain slices of juvenile and adult mice. Our tailored MEA- and spike sorting-protocols allowed high throughput and long recording times. According to individual dopamine-responses, we identified two distinct SN cell-types, in similar frequency: dopamine-inhibited and dopamine-excited neurons. Dopamine-excited neurons were either silent in the absence of dopamine, or they displayed pacemaker-activities, similar to that of dopamine-inhibited neurons. Inhibition of pacemaker-activity by dopamine is typical for SN DA neurons, and it can undergo prominent desensitization. We show for adult mice, that the number of SN DA neurons with desensitized dopamine-inhibition was increased (~60–100%) by a knockout of NCS-1, or by prevention of NCS-1 binding to D2-autoreceptors, while time-course and degrees of desensitization were not altered. The number of neurons with desensitized D2-responses was also higher (~65%) at high glucose-levels (25 mM), compared to lower glucose (2.5 mM), while again desensitization-kinetics were unaltered. However, spontaneous firing-rates were significantly higher at high glucose-levels (~20%). Moreover, transient glucose-deprivation (1 mM) induced a fast and fully-reversible pacemaker frequency reduction. To directly address and quantify glucose-sensing properties of SN DA neurons, we continuously monitored their electrical activity, while altering extracellular glucose concentrations stepwise from 0.5 mM up to 25 mM. SN DA neurons were excited by glucose, with EC_50_ values ranging from 0.35 to 2.3 mM. In conclusion, we identified a novel, common subtype of dopamine-excited SN neurons, and a complex, joint regulation of dopamine-inhibited neurons by dopamine and glucose, within the range of physiological brain glucose-levels.

## Introduction

Dopamine and dopamine-releasing (DA) neurons are important for a variety of brain functions and processes like movement control, habit-formation, conditioning, cognition, novelty-related behavior, motivation, reward prediction, and glucose homeostasis (Dodson et al., 2016; Koekkoek et al., 2017; Berke, 2018; Ter Horst et al., 2018; Collins and Saunders, 2020). DA midbrain neurons in the Substantia nigra (SN) are particularly important for voluntary movement control, and their progressive degeneration causes the main motor-symptoms of Parkinson's disease (PD), the 2nd most common neurodegenerative disease (Kordower et al., 2013; Obeso et al., 2017; Poewe et al., 2017; Hernandez et al., 2019). SN DA neurons are particularly vulnerable to degenerative stressors, while neighboring DA neurons in the ventral tegmental area (VTA) are less affected (Fu et al., 2016; Vogt Weisenhorn et al., 2016; Surmeier et al., 2017). The causes for this differential vulnerability of DA neurons and for their selective degeneration in PD are still unclear. However, a variety of intrinsic and extrinsic factors have been identified, pointing to converging mechanisms, in particular cell-specific electrical activity patterns, calcium homeostasis and elevated metabolic stress (Duda et al., 2016; Giguere et al., 2018; Gonzalez-Rodriguez et al., 2020; Lebowitz and Khoshbouei, 2020).

SN DA neurons display a high level of intrinsic metabolic stress already under control conditions. This is due to the size and complexity of their unmyelinated axonal arbors in their striatal target regions, which is an order of magnitude greater than that of less susceptible dopamine neurons (Bolam and Pissadaki, 2012; Giguere et al., 2019; Wong et al., 2019), as well as their particular mode of electrical activity. SN DA neurons display an autonomous pacemaker activity that is crucial for axonal and somatodendritic dopamine release (Rice and Patel, 2015; Sulzer et al., 2016; Liu and Kaeser, 2019). This activity causes additional metabolic stress, as it is associated, in SN but not in VTA DA neurons, with oscillatory elevated levels of free intracellular Ca^2+^, due to the activation of voltage-gated Ca^2+^ channels (Cav) during action potentials (Zamponi et al., 2015; Philippart et al., 2016; Liss and Striessnig, 2019; Zampese and Surmeier, 2020). These activity-related Ca^2+^ dynamics stimulate electrical activity, ATP synthesis and dopamine release, and thus facilitate movement. But they also constitute an intrinsic metabolic burden that can trigger neurodegeneration. Consequently, the activity of SN DA neurons is tightly controlled by a variety of (still not fully understood) feed-back and feed-forward mechanisms (Dragicevic et al., 2015; Duda et al., 2016; Michel et al., 2016; Gantz et al., 2018).

Dopamine itself is modulating the activity of SN DA neurons in a negative feedback-loop by activation of inhibitory dopamine-autoreceptors of the D2-type (D2-AR) (Lacey et al., 1987; Beckstead et al., 2004; Ford, 2014). Somatodendritic D2-AR are best-known to activate G-protein coupled inwardly rectifying K^+^ channels (GIRK2), and leading to hyperpolarized membrane potentials, but other signaling mechanisms are described (Cardozo and Bean, 1995; Evans et al., 2017; Philippart and Khaliq, 2018; Chen et al., 2020). Inhibitory dopamine responses can desensitize, meaning that neuronal activity is regained although dopamine is still present (Gainetdinov et al., 2004). D2-receptor desensitization is modulated by many (incompletely characterized) processes (Bonci and Hopf, 2005; Robinson et al., 2017a). In particular by Ca^2+^ dependent processes (Beckstead and Williams, 2007; Liu et al., 2008; Perra et al., 2011; Evans et al., 2017; Gantz et al., 2018), like the Ca^2+^ dependent binding of the neuronal Ca^2+^ sensor NCS-1 to D2-ARs, as demonstrated for SN DA neurons from juvenile mice (Kabbani et al., 2012; Dragicevic et al., 2014; Poetschke et al., 2015). NCS-1 is of special interest as it stimulates mitochondrial function and promotes neuronal survival (Catoni et al., 2019; Nakamura et al., 2019). In SN DA neurons, NCS-1 regulates the expression of genes involved in mitochondrial function, and NCS-1 loss increased their vulnerability in a PD mouse model (Benkert et al., 2019; Simons et al., 2019). In remaining human SN DA neurons from *post-mortem* PD brains, NCS-1 mRNA levels are elevated (Dragicevic et al., 2014), pointing to a neuroprotective role of NCS-1 in PD. Dopamine, D2-receptors, and NCS-1 are also important for regulation of glucose homeostasis, pancreatic insulin secretion, and body weight (Gromada et al., 2005; Lopez Vicchi et al., 2016; Hassanabad and Fatehi, 2019; Ratai et al., 2019; Liu et al., 2020). SN DA neurons and pancreatic beta-cells (responsible for peripheral glucose sensing and insulin secretion) modulate each other's function and possess similar signaling mechanisms, like D2-receptors, K-ATP channels, and Cav mediated activity-modulation (Eberhard, 2013; Fiory et al., 2019; Farino et al., 2020; Liu et al., 2020). However, direct glucose sensing properties of SN DA neurons have not yet been reported. Glucose sensing neurons are best described in the hypothalamus, but also in other brain regions (Koekkoek et al., 2017; Alvarsson and Stanley, 2018). They alter their electrical activity with changes in extracellular glucose levels. Glucose-excited (GE) neurons increase their action potential frequency as glucose levels increase (similar to pancreatic beta cells), whereas glucose-inhibited (GI) neurons decrease their activity with rising glucose levels (Routh, 2010).

Here, we studied the modulation of SN DA neuron activity by dopamine, NCS-1, and glucose. As this analysis is complicated by the particularly high vulnerability of SN DA neurons toward metabolic stressors, we used optimized extracellular multi-electrode array (MEA) approaches to study neuronal activity in vital mouse brain slices. MEA recordings followed up by spike-sorting allowed us to analyze SN DA neurons without selection-bias and with much higher throughput compared to patch clamp techniques. Based on their dopamine-responses, we identified two types of SN neurons that were either dopamine-excited or dopamine-inhibited. Both SN populations were similar in size. The number of dopamine-inhibited SN DA neurons with desensitized responses was reduced by NCS-1 activity, and increased by elevated extracellular glucose, but the kinetics of inhibitory dopamine-responses were not altered. SN DA neurons displayed fast and reversible glucose-responsiveness in a dose-dependent manner, with higher electrical activity at elevated glucose levels, and within the physiological range of brain glucose levels. Thus, SN DA neurons display GE-neuron properties, similar as beta-cells.

## Materials and Methods

### Animals

All mice were bred and housed in the facilities of the University of Ulm, according to the German Tierschutzgesetz and the directive 2010/63/EU. All animal procedures were approved by the Regierungspräsidium Tübingen (Reg. Nr. 0.147 and TV1043). Only male mice were analyzed. Wildtype mice are derived from C57BL/6J. NCS-1 KO mice (obtained by Olaf Pongs) were back-crossed at least 10 times into C57BL/6J (Ng et al., 2016), leading to a 99.9% analogy with C57BL/6J, and losing the 129/SvJ original background (Eisener-Dorman et al., 2009). Data from littermate NCS-1 +/+ were not significantly different from those of C57BL/6J and were pooled.

### Patch Clamp Experiments

Coronal mouse brain slices were prepared (similar as detailed below for MEA experiments), and perforated patch clamp recordings were performed and analyzed, as previously described (Dragicevic et al., 2014; Poetschke et al., 2015).

### Multi-Electrode Array (MEA) Experiments

#### Brain Slice Preparation

Juvenile and adult mice were deeply and terminally anesthetized with isoflurane, and decapitated. During slicing, for orientation, a tiny hole was punched into the left side of the brain using a blunted 21G cannula.

For juvenile mice (~PN 13), after decapitation, the brain was quickly removed from the cranial cavity, cooled and cut into 250 μm coronal midbrain slices in ice cold artificial cerebrospinal fluid (ACSF) using a vibratome (Vibroslice^TM^ type: HA752, Campden Instruments, UK), and stainless steel blades (Campden Instruments Ltd.). ACSF contained (in mM): 125 NaCl, 2.5 KCL, 25 NaHCO_3_, 1.25 NaH_2_PO_4_, 2 MgCl_2_, 2 CaCl_2_, and 25 glucose, bubbled with carbogen (95% CO_2_/5% O_2_) for pH adjustment (7.3) and oxygenation. Slices were allowed to recover for 30 min at room temperature in the same ACSF.

For adult mice, brain slice preparation was carried out utilizing a slightly altered version of the “slice it hot” protocol (Ankri et al., 2014). After brain removal, coronal midbrain slices (200 μm) were prepared using a vibratome (Leica VT1200S, amplitude 1.4 mm, speed 0.6 mm/s) with ceramic blades (Campden Instruments Ltd.) in ACSF at ~36°C. This ACSF contained (in mM): 124 NaCl, 3 KCl, 26 NaHC0_3_, 1.2 KH_2_PO4, 1 MgCl_2_, 1 CaCl_2_, 10 glucose, bubbled with carbogen (95% CO_2_/5% O_2_) for pH adjustment (7.3) and oxygenation. Slices were allowed to recover for 1 h at 37°C in ACSF-recovery solution containing (in mM): 125 NaCl, 2.5 KCl, 25 NaHCO_3_, 1.25 NH2PO_4_, 1 MgCl_2_, 1 CaCl_2_, 25 mM glucose.

All chemicals were obtained from Sigma, if not stated otherwise.

#### MEA Recordings

The ACSF for MEA-recordings (“recording-ACSF”) contained (in mM): 125 NaCl, 2.5 KCl, 25 NaHCO_3_, 1.25 NH_2_PO_4_, 1 MgCl_2_, 1 CaCl_2_, with variable glucose concentrations (0.5–25 mM). We added 10 μM DNQX disodium salt (Tocris) and 10 μM Gabazine (Tocris) to block fast synaptic transmission. When glucose was below 25 mM, sucrose was added to get a total of 25 mM sugar in the solution to keep the osmolarity stable (300–315 mOsm/l). ACSF was bubbled with carbogen (95% CO_2_/5% O_2_) for pH adjustment (7.3) and oxygenation.

For MEA-recordings 3D-Biochips were used, manufactured by Qwane Biosciences SA (Switzerland). The electrode array layout of the MEA-chips was customized for covering the shape of the SN in coronal mouse brain sections. The MEA-chips contain 60 3D tip-shaped platinum electrodes, arranged in a 15 row × 4 column array. Interelectrode distance is 100 μm in rows and 90 μm in columns (measured from center to center). Each electrode is 30 μm in diameter, and the tip extends 25–40 μm from the base. The surface of the chips is covered by a 5 μm layer of SU-8 epoxy and has an electrical impedance of 500–800 kΩ. For optimized tissue to electrode contact, biochips were plasma cleaned every second week for 2 min under low vacuum (0.5 Torr, service of the Electron Microscopy Facility, University of Ulm). MEA chips were stored at 4°C, and reused for 3–6 months depending on the intensity of usage.

**Figure 1:**
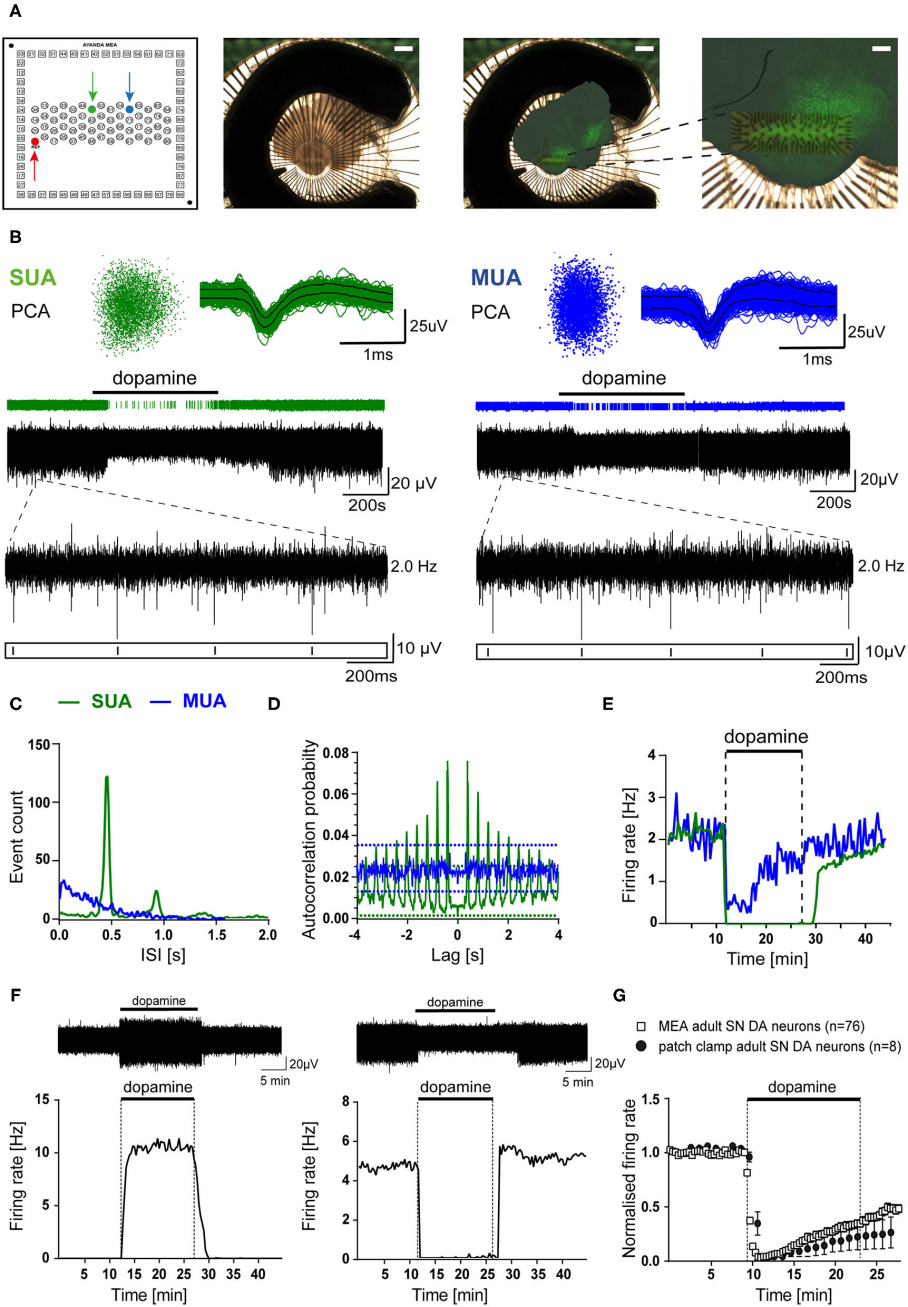
MEA analysis identified dopamine-inhibited and dopamine-excited *Substantia nigra* neurons in mouse midbrain slices. Recordings were obtained from C57BL/6J mouse brain slices in ACSF containing 2.5 mM glucose **(A)** Left panel: schematic channel layout of the used 3D MEA chip, containing 60 platinum 3D-electrodes. The red arrow marks the reference-electrode. The electrodes marked by green and blue arrows correspond to the respective recordings shown in **(B–E)**. Middle panel: Image of a typical coronal midbrain slice, mounted on the 3D MEA biochip (scale bar: 1 mm). Right panels: Overlay of the image before recording with the respective image of the same slice, fixed and stained for tyrosine hydroxylase (TH), after MEA-recording. Magnification (far right) allows identification of the electrode array positioning within the SN (scale bars: 1 mm, and 250 μm, respectively). **(B)** Spike sorting analysis of MEA recordings from two individual electrodes (marked in **A**, left), with identified single unit activity (SUA, left) and multiunit activity (MUA, right) with inhibitory dopamine-responses (bath-application of 100 μM dopamine indicated by horizontal bars). Upper panel: 3D-spike templates after principal component analysis (PCA, left) and overlaid spike-waveforms (right). Lower panel: event-plots and original traces over time. Inserts display enlargements of 2 s, as indicated. **(C)** Histograms (event counts) of individual interspike-intervals (ISI, in 20 s bins) for the two electrodes from **(A,B)**. Note one clear peak and no activity below 200 ms, only in the SUA. **(D)** Autocorrelation probabilities, plotted against ISI (using a Boxcar filter smoothing routine of three bins in width). Note that only the SUA shows clear regular activity exceeding the 99% confidence interval. **(E)** Firing rates (in Hz) plotted over time (in 20 s bins) for both recordings. Note fully inhibited activity by dopamine, typical for SN DA neurons, only for the SUA. **(F)** Upper panels: Exemplary SUA MEA-recording traces. Lower panels: Firing rates plotted over time (in 20 s bins) of a dopamine-excited (left) and a dopamine-inhibited (right) SN neuron (bath application of 100 μM dopamine as indicated). **(G)** Comparison of mean normalized firing rates of dopamine-inhibited SN DA neurons from adult mice, recorded with MEA (white squares), or with perforated-patch clamp techniques (black circles). Shown are normalized mean frequencies, plotted over time (20 s bins for MEA, 1 min bins for patch clamp; MEA: *n* = 76, perforated patch: *n* = 8). Data are given as mean ± SEM. All data and statistics are detailed in Supplementary Figures 1, 4A and Supplementary Tables 1, 2.

MEA recordings were carried out using either a USB MEA1060-Up-BC System or a MEA2100-2x60 System (Multichannel Systems, Germany). For the MEA1060-System, slices were mounted onto the MEA-chip under a stereoscope (Leica M165C). For the 2100 MEA System, slices were directly mounted on the amplifier with help of a video table (MEA-VMTC-2, Multichannel Systems). Slices were kept in place by a self-made grid, consisting of nylon strings affixed to a semi-circle platinum wire (3 mm diameter of the wire, 1.3 g total weight). Careful and correct positioning of the slice on the chip and placing the grid without moving or harming the slice was crucial for successful experiments. Before and after placing the grid, images were taken to document the MEA chip positioning (Figure 1).

The recording chamber containing the MEA-chip was constantly perfused with the carbogenated recording-ACSF by a peristaltic pump (~4 ml/min, Ismatec, Germany). The temperature control of the inlet cannula (TC02 with PH01, Multichannel Systems, Germany) was set to 35°C and the inbuilt amplifier heating plate to 40°C, resulting in a bath temperature of 31–33°C (checked by a hand-thermometer before each experiment). After transfer into the recording chamber, slices were allowed to equilibrate in recording-ACSF for 30 min.

Data were sampled at 20 kHz using the MC_Rack software (version 4, Multichannel Systems, Germany). Data was recorded using a low pass (3,000 Hz) and a high pass (200 Hz) butterworth 2nd order filter. The software allows simultaneous recordings and online analysis. The so-called spike analyzer tool was used to identify the electrodes displaying activity directly during recordings: This tool is an in-built spike sorter that detects spikes within the signals, recorded by each electrode. The threshold for spike detection is set automatically by the software and individually for each electrode depending on the signal to noise ratio. Detected spikes were continuously displayed in the spike analyzer display. Electrodes showing a reaction to DA application (i.e., change of event-rate, either elevated or reduced) were marked for further data analysis (see below).

The basic experimental design, carried out in recording-ACSF, was as follows (with 2.5 mM glucose if not stated otherwise): 10 min baseline recording of pacemaker-activities (for determining basal frequency and CV-ISI-values), followed by bath-application of 100 μM dopamine hydrochloride for 15 min, and a 20 min wash-out phase of dopamine. For DNIP (D2R/NCS-1 interaction prevention) experiments the DNIP peptide, or its scrambled (srDNIP) control, were synthesized by Genscript according to the published amino acid sequences (Saab et al., 2009; Kabbani et al., 2012). Peptides were dissolved in recording-ACSF, and bath-applied at a final concentration of 10 μM. DNIP-experiments were carried out as follows: after 10 min baseline recordings, slices were incubated for 30 min with recording-ACSF containing DNIP/srDNIP, followed by recording of 10 min baseline activity, 15 min dopamine application, and 20 min washout of dopamine (all in recording-ACSF with DNIP/srDNIP). For glucose sensing experiments, different glucose concentrations were bath-applied by switching recording-ACSFs with respective different glucose concentrations. Delay-time until dopamine/peptides/altered glucose reached the recording chamber was about 120 s. For NCS-1 experiments, results for adult mice (~PN90) are shown, but SN DA neurons from juvenile mice displayed similar responses (data not shown; and see Dragicevic et al., 2014; Poetschke et al., 2015). For glucose sensing experiments, results for juvenile mice are shown (~PN13) as they coped better with very low glucose concentrations, but SN DA neurons from adult mice displayed similar qualitative glucose responses.

### Data Analysis

#### Spike Sorting

MEA-recording files were saved as mcd-files. Electrodes that recorded a response to dopamine (i.e., change of event-rate) were already marked during the recording, based on the online analysis options in the MC_Rack software. To minimize file sizes, recording files were re-recorded containing only the electrodes displaying a response to dopamine (either increased or decreased event-rate). The generated second mcd file was imported into Spike2 and converted to a smrx file.

Spike sorting of MEA recordings was carried out with Spike2 software (8.02e x64, Cambridge Electronic Design Ltd.). A threshold trigger was manually set by the experimenter, depending on the signal to noise ratio of the respective recording. The detected spikes displayed an amplitude of ~25-50 μV with a background noise of 15-25 μV. Via principal component analysis (PCA), Spike2 generates so-called spike templates, by matching and grouping spikes according to their principal components. These principal components are distinct features of the spikes like shape, amplitude, and length. The algorithm tries to separate the spikes in a direction of the largest variance. Meaning that the principal components with the highest variation in-between are chosen to generate the final spike templates. Spikes were separated by the software choosing the first two or three principal components (Lewicki, 1998). Templates were then automatically plotted in a 3D coordinate system as clusters, where every spike is displayed as a single dot and the chosen principal components represent the three axes x, y, and z. This enables manual reassignment of spikes, if necessary. For further details see manual “Spike2 version 9 for Windows.”

#### Identification of Single Unit Activity (SUA) With Spike2 Software

If spike sorting by automatic Spike2 analysis was successful, the activity will be split into separate single unit activities. This means, that each activity, recorded from the individual electrodes, can be assigned to a single neuron. Since this was not always the case, after the automatic principal component analysis by Spike2 software, manual cluster analysis optimization by the experimenter is often necessary, to sort out appropriate SUAs out of automatically classified multi unit activity (MUA).

For identification of a single unit activity (SUA), the sorted activity was manually checked in Spike2 for the following pre-selection criteria: (a) a lack of events occurring during the first 200 ms (short ISI range) shown in an interval histogram: the number of events before the first big peak of the interval histogram must not exceed 15% of this peak. (b) A clear and regular pacemaker activity, belonging to one unit, as judged by autocorrelation. The autocorrelation is defined as the correlation of the activity of a unit with a delayed copy of itself. (c) A lack of cross-correlation with other units recorded from the same electrode (for multi unit activity, only). (d) The appropriate typical shape of the waveform average of the unit. Supplementary Figure 1 shows a typical example of a SUA, derived from a single SN DA neuron. Only units fulfilling these criteria were further analyzed. Clusters representing obvious multi unit activity, meaning that they very likely contain activities of more than one neuron, were excluded from further analysis. These criteria are used only for pre-selection of possible SUAs. Statistical analysis is carried out subsequently with Neuroexplorer, as the options offered by Spike2 are limited, but pre-selection is carried out to reduce the amount of data that has to be imported to Neuroexplorer later.

#### Verification of Single Unit Activity With Neuroexplorer Software, Exclusion of Multi Unit Activity (MUA), and of Duplicate Recordings

After Spike2 analysis, smrx files containing traces, including timepoints of each spike of potential SUAs, were imported into the Neuroexplorer software (vers. 4.032, Nex Technologies, USA) to further analyze spontaneous activities of all recorded units for all electrodes. For this, autocorrelation as well as cross-correlation probability histograms were generated and analyzed. As before, autocorrelation histograms correlate a unit with a delayed copy of itself, while cross-correlation analysis allows to identify if the same SUA is recorded by more than one electrode, as all units recorded on one slice are analyzed for correlation. For both, 10 min of baseline activities were analyzed using a Boxcar filter smoothing routine of 3 bins (30 ms) in width (Berretta et al., 2010). Auto-correlation probability had to exceed the 99% confidence interval to be identified as SUA and included into the dataset. All identified SUAs, recorded with the same MEA-chip were tested for synchrony via their cross-correlation probability plots. In case units display a peak cross-correlation probability exceeding the maximal 99% confidence interval, the electrode with the better sorted signal (better signal to noise ratio) was included, the other was excluded from the dataset, as this high correlation is typical if the same neuron had been recorded from two different electrodes (Berretta et al., 2010). However, we cannot exclude that this approach occasionally eliminates SUAs from different cells with highly synchronized firing-patterns.

#### Further Analysis of Verified SUA

For further analysis, the mean firing rate (in 20 s bins) of each verified SUA was exported into excel from Spike2. To define the basal firing rate, the mean firing rate of a stable 10 min control period at the beginning of each recording was calculated. For describing pacemaker-regularity, the mean interspike-interval (ISI = 1/frequency) and its standard deviation (SD) were determined for this 10 min control period, to calculate the coefficient of variation (CV) of the ISI:

$$\begin{array}{l} CV\;\left[\%\right]=\;\frac{SD\;\left(ISI\right)}{mean\;\left(ISI\right)}*\;100 \end{array}$$

Cells displaying a pacemaker frequency above 6 Hz or CV-ISI values higher than 30% were excluded from further analysis in all data-sets, as typical SN DA neurons in mouse brain slices in synaptic isolation display slower and very regular pacemaker activities in the range of ~1–4 Hz with CV-ISI between about 5 and 15% (Wolfart and Roeper, 2002; Lammel et al., 2008; Poetschke et al., 2015). VTA DA neurons display a faster (still under 10 Hz) and a less regular pacemaker (Lammel et al., 2008; Khaliq and Bean, 2010; Morales and Margolis, 2017). Note that MEA-derived CV-ISI values can differ from those derived from patch clamp experiments, due to the automated spike-sorting process and missed spikes.

For analysis of relative firing-rates in dopamine, firing-rates in the last minute in dopamine (i.e., minute 15) were normalized to the mean firing rate during the 10 min control period. An inhibitory dopamine D2-AR response was classified as desensitized if the mean frequency in the last minute of dopamine was higher than 5% of the respective basal firing rate.

Cumulative glucose dose-response curves were fitted and EC_50_ values were determined by using the hill equation (in PRISM, equation type: log(agonist) vs. response-variable slope, according to Goutelle et al., 2008):

$$\begin{array}{l} \text{y}=\text{ bottom}+\;\frac{{\text{x}}^{\text{hillslope}}\;*\;\left(\text{top }-\text{ bottom}\right)}{{\text{x}}^{\text{hillslope}}+{\text{E}{\text{C}}_{50}}^{\text{hillslope}}} \end{array}$$

With y = normalized firing rate, x = glucose concentration, hillslope = steepness of the curve, top = upper plateau of the curve, bottom = lower plateau of the curve. Top and bottom were set to 1 and zero, respectively. The hillslope was freely fitted. EC_50_ values are given as the mean of individual EC_50_, derived from individual fits for each analyzed cell (mean of individual fits), as well as from the fit of the mean values (fit of means, population analysis).

#### Statistical Analysis

Data are given as mean ± standard error of mean (SEM) if not stated differently. Standard Error (SE) or Standard Deviation (SD) are given as indicated. The number of identified SUAs (i.e., individual cells) is given by n, number of mice is given by N. Data were probed for normal distribution by using the Shapiro-Wilk normality test. As some datasets were not normally distributed, for most comparisons, non-parametric tests were used for probing for significant differences. For statistical comparisons of basal firing rate, CV-ISI and relative activities at minute 15 in dopamine, a two-tailed, unpaired, non-parametric Mann-Whitney-test (for pair-wise comparisons) was used. Differences in the ratios of sensitized to desensitized D2-AR responses were assessed by chi-square test for pair-wise group comparisons. To evaluate differences in firing rates over time, two-way repeated-measures ANOVA (with Sidak's multiple comparison *post-hoc* test) was used. For comparison of mean firing rates and CV-ISI in different glucose concentrations, repeated-measures one-way ANOVA tests were used (Friedman test with Dunn's multiple comparison *post-hoc* test). For comparison of mean firing rates, CV-ISI and relative activities at minute 15 of DA between NCS-1 KO/WT and DNIP/srDNIP as well as DA-excited and -inhibited SN neurons unpaired Kruskal-Wallis tests with Dunn's multiple comparison were used. To detect differences in the change of firing-rate and the CV-ISI between decrease and increase of glucose concentration the Wilcoxon matched-pairs signed rank test was used. A single asterisk (^*^) denotes a *p* < 0.05, two (^**^), three (^***^), and four asterisks (^****^) denoting *p* < 0.01, 0.001, and 0.0001, respectively. All statistical analysis and data transformations were performed in Prism (GraphPad Software Inc., USA).

### Immunohistochemistry and Anatomical Maps

To reconstruct the position of the electrodes, after MEA recording, slices were immunostained for tyrosine hydroxylase (TH), similar as previously described (Lammel et al., 2008; Benkert et al., 2019). Therefore, after recordings, the grid was removed carefully using a forceps, and slices were transferred, via a suction pipette, into a small glass containing recording ACSF. Slices were immediately fixed with 4% PFA (Thermofisher Scientific) in 1 × PBS (phosphate buffered saline, Thermofisher Scientific, tablets, dissolved according to instructions) for 1 h at room temperature. Slices were then transferred into a storing solution containing (in %): 0.05 sodium azide, and 99.95 PBS, and stored at 4°C. Slices were washed three times (10 min each) with PBS, and kept on a microplate shaker (300 rpm, VWR). Slices were then incubated in blocking solution containing (in %): 10 goatserum (Vector Labs), 0.2 BSA (Roth), 0.5 Triton X in PBS, for 2 h to prevent unspecific binding of the antibody. Afterwards, slices were washed one time (10 min) with PBS followed by an overnight incubation with the primary antibody (1: 1000, mouse anti-TH, Merck Millipore). Slices were then washed three times for 10 min with PBS, followed by incubation with the secondary antibody (1:1000, Alexa Fluor 488 goat anti-rabbit, Thermofisher Scientific) for 3 h at room temperature, under light protection, while shaking. The following steps were performed in darkness using tinfoil to cover the well plates: The slices were washed three times for 10 min with PBS. Afterwards, the slices were mounted on superfrost glass slides (VWR), dried for 10 min, and covered with Vectashield Antifade Mounting Medium (Vector Laboratories, United States), and stored in the dark at 4°C. Images were taken with an epifluorescent microscope (Leica DM6500 microscope with a Leica DFC7000 T camera), and documented with LAS-X software.

Graphical overlays of images taken before the recordings and of images after TH-staining were created using the GNU Image Manipulation Program (GIMP vers. 2.8.14). Neuroanatomical positioning in maps was based on a mouse brain atlas (Paxinos and Franklin, 2001).

### Data Availability

All datasets presented in this study are available from the corresponding author upon request.

## Results

### MEA Analysis Identifies Dopamine-Inhibited and Dopamine-Excited *Substantia nigra* Neurons in Mouse Midbrain Slices

To allow a comprehensive analysis of neuronal activity patterns, without compromising intracellular signaling and with high throughput, we established a suitable protocol for extracellular brain slice recordings using multi-electrode arrays (MEA). The utilized MEA-chip (custom-build by Qwane Biosciences SA) contained 60 3D tip-shaped platinum electrodes (30 μm tips), arranged in a 15 row × 4 column array (Figure 1A). Coronal midbrain slices were mounted on these chips, so that the electrodes were covering the SN region, and chip positioning was documented. For verification of correct positioning of the electrodes within the SN, after recordings, slices were PFA-fixed and stained for tyrosine hydroxylase (TH), a marker for DA neurons in the midbrain (Figure 1A right panels). Electrodes located outside of the TH-positive SN, according to picture-overlays, were excluded from further analysis.

Electrodes can record activity from more than one neuron (multi unit activity MUA), but only electrodes with identified single unit activity (SUA) were further analyzed. SUA and MUA were identified after spike-sorting and principal component analysis (PCA). Figure 1B shows examples of a typical SUA and MUA, derived from SN DA neurons. MUA were discriminated from SUA by a high number of events in the range before 200 ms (Figure 1C; >15% of big peak), an irregular pacemaker activity, assessed by a higher variation of the inter-spike interval (ISI, Figure 1D), and an only partial inhibition by dopamine (100 μM, bath-applied for 15 min; Figure 1E). These criteria can be used for identifying SUA derived from SN DA neurons, as they display *in vitro* in synaptic isolation a slow, very regular pacemaker-activity (~0.5-5 Hz, CV ISI: ~5–10%), and a well-described full inhibition of pacemaker-activity in response to extracellular dopamine (Lacey et al., 1987; Mercuri et al., 1994; Beckstead et al., 2004; Lammel et al., 2008). SUA derived MEA recordings from SN DA neurons displayed a clear peak in the event count histogram, low number of events in the 1-200 ms range, an autocorrelation probability exceeding the 99% confidence interval (CI), and a full inhibition of spontaneous activity by dopamine (Figures 1C–E, Supplementary Figure 1). To identify and exclude recordings of the same neuron from more than one electrode, cross-correlation histograms were generated for all electrodes on one slice. For electrodes displaying a cross-correlation probability exceeding the 99% confidence interval, the one with lower signal was excluded (Supplementary Figure 1C). We excluded about 4 ± 1 electrodes as presumed MUA per recorded slice, and less than one in 10 slices based on the cross-correlation analysis. With this MEA recording and data analysis approach, we detected about 5 ± 1 SN neurons per brain-slice that showed a response to dopamine (~5 lower activity, ~3 higher activity, 33 experiments).

Electrodes that did not respond to dopamine with altered event-rates were not further analyzed, but we analyzed all electrodes that displayed responses to dopamine (higher or lower event-rate). Interestingly, we detected a large population of SUAs that were excited by dopamine (Figure 1F, Supplementary Table 1). We never saw these dopamine-excited neurons in our data-sets from perforated patch clamp or on-cell recordings, while pacemaker activity and responses of the dopamine-inhibited SN neurons, recorded with perforated patch clamp recordings, were not significantly different from those recorded with our MEA approach (Figure 1G, Supplementary Figure 4A, Supplementary Table 2) (Liss et al., 2005; Lammel et al., 2008; Dragicevic et al., 2015; Poetschke et al., 2015).

**Figure 2:**
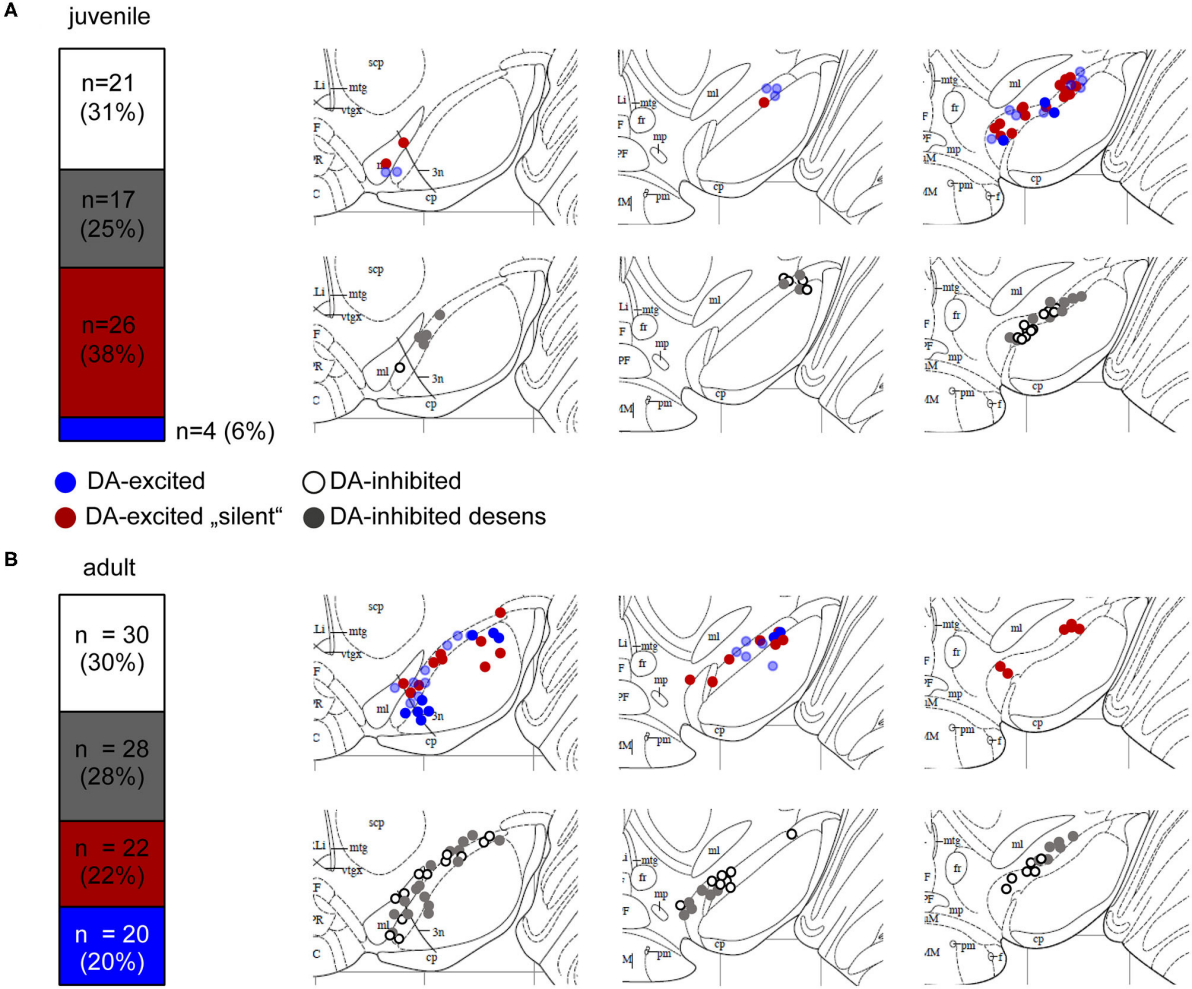
Mouse SN neurons displayed four different types of dopamine responses. Relative frequencies of four identified different dopamine-responses, and anatomical locations of MEA-recorded neurons from juvenile **(A)** and adult **(B)** mice. Experiments, MEA recordings, and data analysis described in Figure 1. Data were derived from juvenile (~PN13) and adult (~13W) C57BL/6J mice, recorded in ACSF containing 2.5 mM glucose. Left panels: SN neurons were classified according to their dopamine responses in DA-excited (44%, juveniles, 42% adults) and DA-inhibited (56% juveniles, 58% adults) cells. DA-excited cells were subclassified in neurons with (blue) and without (“silent,” red) spontaneous activity before DA-application. DA-inhibited cells were subdivided in neurons with (“desens,” grey) and without (white) prominent desensitization of dopamine responses over time. Numbers of DA-excited neurons with activity before DA-application was significantly higher in adults (*p* = 0.0025). Right panels: Maps displaying the anatomical locations of the DA-excited (upper) and DA-inhibited (lower) neurons within the SN, on caudal (left), medial (middle), and rostral (right) coronal slices. Maps are derived form the Paxinos mouse brain atlas (reproduced with permission from Elsevier Ltd.), figures 56, 57, 60 (Bregma: −3.08, −3.16, −3.52), respectively (Paxinos and Franklin, 2001). All data and statistics are detailed in Supplementary Table 1.

With MEA recordings, we robustly identified DA-excited SN cells in juvenile and adult mice, in a similar frequency as we detected DA-inhibited cells (Figure 2, Supplementary Table 1, juveniles: 44% of all dopamine-responsive cells; ~3 cells/slice; *n* = 30 from 11 slices, *N* = 7 mice; adults: 42% of all dopamine-responsive cells; 3 cells/slice; *n* = 42 from 14 slices, *N* = 7 mice). In most recordings from juveniles (87%, *n* = 26 of 30), and in about 50% from adults (*n* = 22 of 42), these dopamine-excited SN cells showed no activity at all before dopamine application but got transiently excited by dopamine (juveniles: 3.39 ± 0.97 Hz, adults: 4.2 ± 2.6 Hz; mean ± SD). The subpopulation of adult dopamine-excited cells that did display pacemaker activity before dopamine-application (3.21 ± 2.99 Hz, mean ± SD) increased its frequency almost 3-fold during dopamine application to 8.8 ± 6.7 Hz (mean ± SD). Pacemaker frequencies in dopamine were significantly higher in neurons that displayed activity before dopamine-application compared to those without activity (about 50%). The baseline pacemaker-activity of dopamine-excited cells compared to that of dopamine-inhibited cells appeared a bit faster but was not significantly different. However, the pacemaker (before dopamine) was less precise in adults (CV-ISI inhibited: 9.70 ± 5.28%, excited: 16.07 ± 10.48%; *p* = 0.0137). We also mapped the anatomical location of dopamine-excited and dopamine-inhibited cells (Figure 2). However, we identified no specific localization of either of these cell-types.

For our further analysis, we focused on dopamine-inhibited SN neurons, as this response is well-described for SN DA neurons. In juveniles and adult, SN DA neurons with inhibitory dopamine-responses displayed prominent desensitization in <50% of analyzed neurons (Figure 2, Supplementary Table 1). Basal pacemaker-frequencies were ~20% higher in SN DA neurons with desensitized D2-AR compared to those with sensitized responses. This difference was significant, when pooled datasets were analyzed (juveniles: sens: 1.54 ± 0.07 Hz, desens: 1.81 ± 0.09 Hz, *n* = 75/141, *p* = 0.027, pooled analysis of WT data from Supplementary Tables 1, 3; adult: sens: 1.64 ± 0.08 Hz, desens: 2.15 ± 0.11 Hz *n* = 50/119, *p* = 0.0005, pooled analysis of WT data from Supplementary Tables 1, 5). In SN DA neurons from juvenile mice, we have previously shown by perforated patch clamp experiments, that this desensitization depends on free intracellular Ca^2+^ and binding of NCS-1 to the D2-AR (Dragicevic et al., 2014; Poetschke et al., 2015). As SN DA neurons from adult mice are even more vulnerable than those of juveniles, we were not successful in addressing desensitization mechanisms with perforated patch clamp experiments.

### The Neuronal Calcium Sensor NCS-1 Reduces the Number of SN DA Neurons With Desensitized Inhibitory Dopamine-Responses by Binding to D2-Receptors

**Figure 3:**
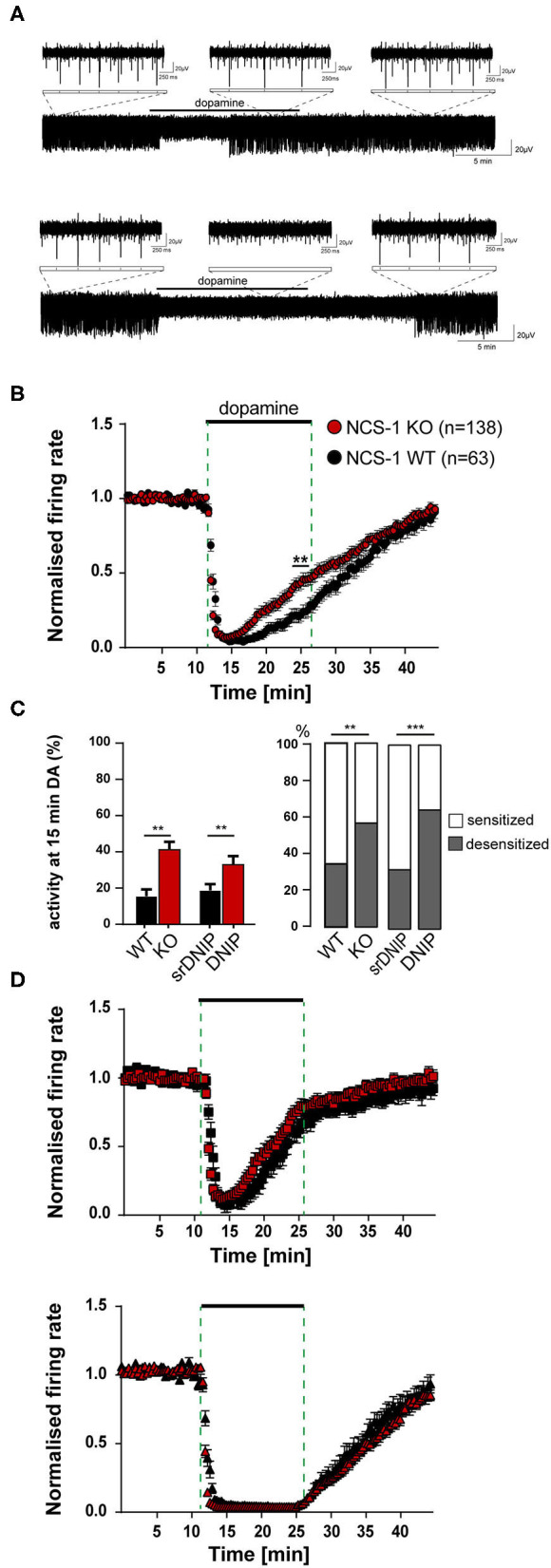
Loss of NCS-1 function increases the number of SN DA neurons with desensitized inhibitory dopamine-responses. Experiments, MEA recordings, data analysis, and presentation similar as in Figure 1. Data were derived from adult NCS-1 WT, NCS-1 KO, and C57BL/6J mice (for DNIP experiments; D2/NCS-1 interacting prevention peptide), recorded in ACSF containing 2.5 mM glucose. **(A)** Exemplary traces of a SN DA neuron with desensitized (upper) and with sensitized (lower) inhibitory dopamine responses (mediated by D2-autoreceptors, D2-AR). Bath application of 100 μM dopamine indicated by horizontal bars. Inserts display enlargements of 2 s during control period, DA application and wash-out phase. **(B)** Normalized mean firing rates plotted over time for all analyzed SN neurons from NCS-1 WT (black, *n* = 63) and NCS-1 KO (red, *n* = 138) mice. **(C)** Left panel: Mean relative firing frequencies in the last minute of dopamine. Right panel: Percentages of cells with desensitized dopamine-responses (grey), for SN DA neurons from NCS-1 WT and KO, and in the presence of 10 μM DNIP (*n* = 64), or 10 μM srDNIP (*n* = 96; scrambled DNIP, as control), as indicated. **(D)** Data from **(B)**, plotted separately for SN neurons with desensitized (upper, WT: *n* = 22, KO: *n* = 77) and with sensitized inhibitory dopamine responses (lower, WT: *n* = 41, KO: *n* = 61). Data are given as mean ± SEM. Significances/*p*-values according to unpaired Kruskal-Wallis test with Dunn's multiple comparison, Chi-square, and unpaired Mann-Whitney-test. All data and statistics are detailed in Supplementary Figures 2, 4B, Supplementary Tables 3, 4.

We used our MEA-approach to address if the presence and the degree of desensitization of dopamine autoinhibition of SN DA neurons from adult mice also depended on NCS-1 binding to D2-AR. We analyzed adult NCS-1 KO mice and wildtype controls. In addition, we analyzed in adult wildtype the responses to a so-called DNIP peptide that prevents NCS-1 binding to D2-ARs, and scrambled DNIP for controls (Figure 3, Supplementary Figures 2, 4B, Supplementary Tables 3, 4). As before, in response to dopamine bath-application, we detected a full and reversible inhibition of pacemaker activity of SN DA neurons from adult wildtype mice, with desensitization of the response in about 65% (Figure 3A, Supplementary Table 4) of recorded neurons. The absence of NCS-1 or the prevention of its binding to the D2-AR in adult SN DA neurons increased the amount of desensitized dopamine-responses, as evident from plotting mean normalized frequencies over time, and from comparing relative activities at the last minute in dopamine (Figures 3B,C, Supplementary Figures 2B,C, Supplementary Tables 3, 4). As the MEA approach allowed us to analyze responses of a large number of neurons, we compared mean basal frequency between neurons with sensitized and desensitized dopamine-responses and analyzed the ratio of the responses (Figures 3C,D, Supplementary Figures 2C,D, Supplementary Tables 3, 4). This analysis revealed that a general knock out of NCS-1, as well as prevention of NCS-1 binding to D2-AR specifically in SN DA neurons, massively increased the number of SN DA neurons displaying desensitized D2-AR responses (about 60% in NCS-1 KO, and over 100% in DNIP). However, the time-course and degree of dopamine inhibition was neither altered by general NCS-1 knock out nor by the DNIP peptide. Hence the observed faster desensitization (Figure 3B, Supplementary Figure 2B) is only caused by higher numbers of SN DA neurons with desensitizing DA-responses and no change in the overall kinetic of the response. Moreover, these results indicate that SN DA neurons might switch between desensitized or sensitized dopamine responses, and that NCS-1 is stimulating sensitized responses.

Sensitized D2-autoreceptor responses by NCS-1 would reduce electrical activity, Ca^2+^ load and metabolic stress in SN DA neurons, while desensitized D2-AR responses would increase dopamine-release and thus facilitate movement—but also elevate Ca^2+^ load and metabolic stress levels. Hence, we hypothesized that general activity and the number of SN DA neurons with desensitized D2-AR responses could be higher at optimal metabolic conditions, and lower in metabolic stress situation. To test this, we analyzed pacemaker activity and dopamine-responses at different glucose concentrations.

### Elevated Glucose Levels Increase the Number of SN DA Neurons With Desensitized Inhibitory Dopamine Responses and Their Pacemaker Firing-Rate

**Figure 4:**
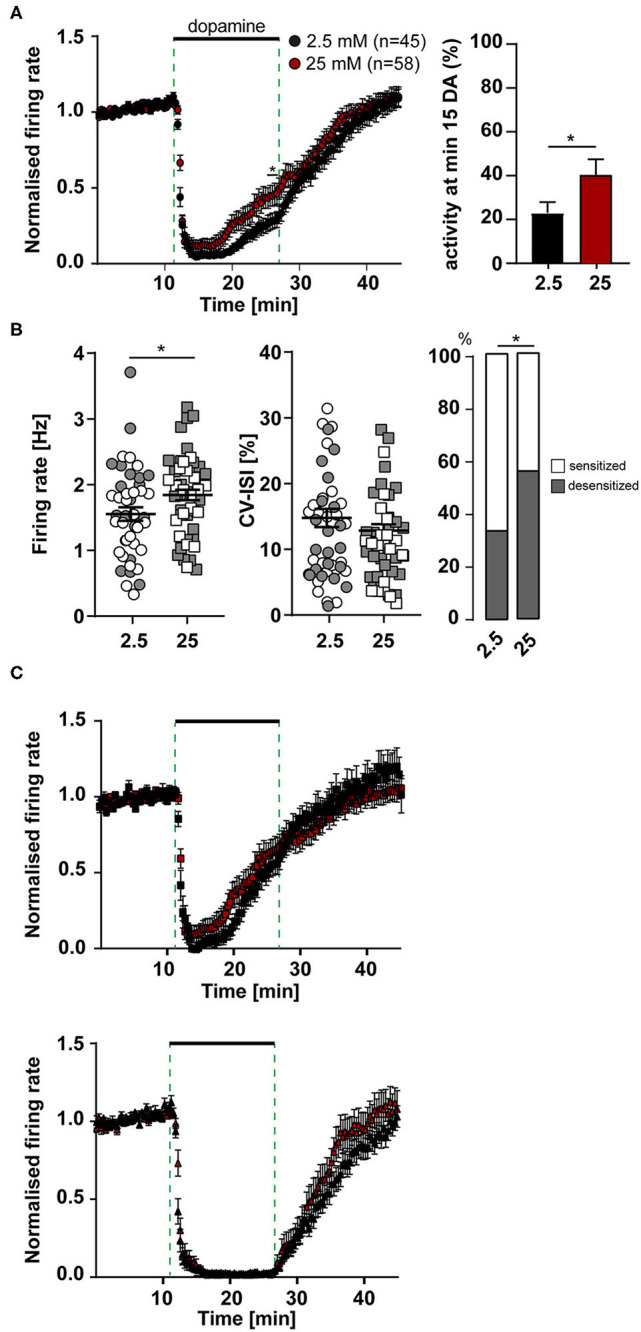
More SN DA neurons with desensitized dopamine D2-AR responses, and with higher pacemaker frequencies in elevated glucose. MEA recordings, data analysis and presentation similar as in Figures 1, 3. Data were derived from juvenile C57BL/6J mice, recorded in ACSF containing either 2.5 mM (*n* = 45) or 25 mM (*n* = 58) glucose. **(A)** Left panel: Normalized mean firing rates of all analyzed SN neurons, plotted over time. Right panel: activity at minute 15 in dopamine. **(B**) Left panels: Mean pacemaker frequencies and pacemaker precision (displayed as CV-ISI), during first 10 min of recordings. Right panel: Percentages of SN DA neurons with desensitized D2-AR responses (grey) in 2.5 and 25 mM glucose. **(C)** Data from **(A)**, plotted separately for SN neurons with desensitized (upper, 2.5 mM glucose: *n* = 20, 25 mM glucose: *n* = 38) and with sensitized inhibitory dopamine responses (lower, 2.5 mM: *n* = 25, 25 mM: *n* = 20). Data are given as mean ± SEM. Significances/*p*-values according to two-way ANOVA with Sidak's multiple comparison, Chi-square and unpaired Mann-Whitney-test. All data and statistics are detailed in Supplementary Tables 5, 6.

We performed similar experiments, as described before, but now comparing activity and the inhibitory dopamine response of SN DA neurons in 2.5 mM glucose with those in 25 mM glucose (Figure 4). In line with our hypothesis, the number of SN DA neurons with a desensitized D2-AR response was significantly higher in 25 mM glucose (~50%, Figures 4A,B, Supplementary Tables 5, 6). Again, the mean time-course and degree of dopamine inhibition was not altered, neither in the group of neurons with sensitized nor desensitized responses (Figure 4C). Moreover, we observed that the mean pacemaker-frequency of SN DA neurons before dopamine application was significantly higher in 25 mM dopamine (~20%; Figure 4A, Supplementary Table 5). Pacemaker precision (CV-ISI) was not different in 25 mM glucose.

These results suggested that SN DA neurons might display glucose sensing properties, similar as pancreatic beta-cells, with higher activity at higher glucose concentrations (termed GE-neurons; MacDonald et al., 2005).

### Transient Glucose Deprivation Reversibly Reduces Pacemaker Activity of SN DA Neurons

To systematically address GE-properties of SN DA neurons, we carried out a set of MEA experiments, where we continuously recorded pacemaker activities of SN DA neurons while changing extracellular glucose levels, by switching recording-ACSF solutions. As glucose sensing neurons have been described in a wide range of glucose concentrations, with so-called high-GE neurons sensing glucose above 5 mM up to over 20 mM (Fioramonti et al., 2004; Routh, 2010; Alvarsson and Stanley, 2018), we chose 25 mM as an optimal glucose concentration, and switched to 1 mM to induce transient glucose deprivation and metabolic stress.

**Figure 5:**
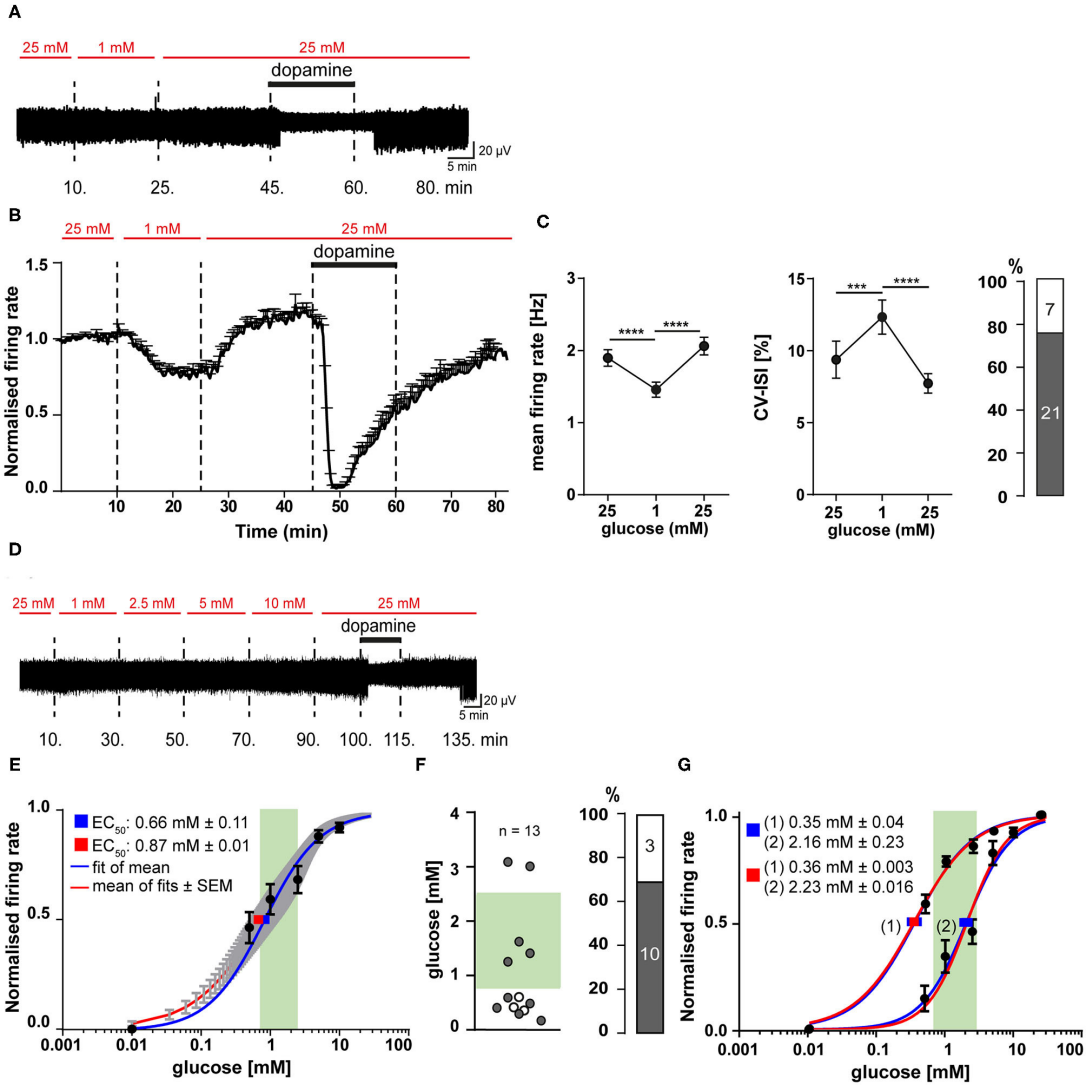
SN DA neurons display fast and reversible glucose-sensitivity in the range of brain glucose levels. Experiments, MEA recordings, data analysis and presentation similar as in Figures 1, 3. **(A)** Exemplary traces, recorded from a mouse SN DA neuron, while glucose was transiently reduced from 25 to 1 mM, as indicated by red horizontal bars. Bath application of 100 μM dopamine indicated by black horizontal bars. **(B)** Normalized mean firing rates of all analyzed SN DA neurons are plotted over time (*n* = 28). **(C)** Left/middle panel: Mean non-normalized firing rates (left) and pacemaker precision (given as CV-ISI, middle) during the first 10 min of the recording in 25 mM glucose, in 1 mM (last 10 min), and back in 25 mM glucose (last 10 min). Right panel: Number/percentage of SN DA neurons from **(B,C)** with desensitized D2-AR responses (grey). **(D)** Exemplary trace, recorded from a mouse SN DA neuron, while glucose concentrations were altered as indicated (0.5–25 mM). Bath application of different glucose concentrations (red) and of 100 μM dopamine (black) indicated by horizontal bars. **(E–G)** Dose-response curves and derived EC_50_ values for all analyzed SN DA neurons (*n* = 13). EC_50_ values were calculated according to the Hill-equation. Values are given as mean ± SE. The range of physiological brain glucose levels is indicated in green, [0.7–2.5 mM], according to Routh et al. (2014). **(E)** EC_50_ values were derived from fitting the mean relative firing rates of all cells at each concentration (blue), and by the mean fit of the fits for each individual neuron (red). Values are given as mean ± SE. **(F)** Left panel: Scatterplot of individual EC_50_ values, derived from individual fits for each cell (gray dots: cells with desensitized D2-AR responses; white dots: cells with sensitized responses). Right panel: Number/percentage of SN DA neurons from **(E)** with desensitized D2-AR responses (grey). **(G)** Separate dose-response curve fits; cells from **(E,F)** were subdivided into neurons with EC_50_ higher (*n* = 5) or lower (*n* = 8) than 1 mM glucose. Data are given as mean ± SEM. Significances/*p*-values according to paired Friedman test with Dunn's multiple comparison and Chi-square test. All data and statistics are detailed in Supplementary Figures 3, 4C,D, Supplementary Tables 7, 8.

As illustrated in Figure 5A, after recording baseline pacemaker activity for 10 min in 25 mM glucose, we switched to 1 mM for 15 min (until frequencies were stable again) and back to 25 mM glucose for another 20 min, followed by application of dopamine (and wash-out), for identification of SN DA neurons. With this paradigm, spontaneous activity of SN DA neurons was reduced in all tested neurons by switch to 1 mM glucose, by about 25% (from a mean of 1.9 to 1.5 Hz), and pacemaking was less precise (~40%; Figures 5B,C, Supplementary Tables 7A,B). Furthermore, we detected with about 75% a high amount of SN DA neurons with desensitized D2-AR responses (Figure 5C, Supplementary Table 7). We also analyzed the effect of transient glucose-deprivation separately for sensitized and desensitized neurons (Supplementary Figures 3A, 4C, Supplementary Table 7). As before, firing rates were higher in SN DA neurons with desensitized D2-AR response (~20%; Supplementary Figures 3A, 4C, Supplementary Table 7).

### SN DA Neurons Display Glucose Sensitivity Within the Physiological Range of Brain Glucose Levels

The transient glucose-deprivation experiments demonstrated for SN DA neurons glucose-sensing properties of the GE-type. To further quantify glucose sensitivity within the (patho-) physiological range of brain glucose levels, we carried out MEA experiments where glucose concentrations were stepwise elevated from 0.5 mM up to 25 mM to determine dose-response curves and EC_50_ values. Experimental design and a typical response of a SN DA neuron is given in Figure 5D. We also tried concentrations lower than 0.5 mM, however, SN DA neurons were not stable enough over the required long recording time (each recording took more than 2 h). To determine half maximal effective (EC_50_) glucose-concentrations for individual SN DA, we fitted dose-response curves for each individual neuron, as well as the mean values at each concentration of all analyzed neurons (population analysis). Both approaches resulted in similar EC_50_ values, slightly below 1 mM glucose (mean ± SE: 0.87 ± 0.01 and 0.66 ± 0.1, *n* = 13 Figure 5E, Supplementary Table 8). As indicated by the high standard deviation (±1.04) and as evident in Figure 5F, the individual EC_50_ values varied substantially and were not homologous distributed (*p* = 0.777). Thus, we separated the analyzed cells into two groups (>1 mM, <1 mM) and fitted them separately. Indeed, the dose-response curves were better fitted by these two fits, than by one over-all fit and resulted in EC_50_ values of 2.23 mM (*n* = 8) and 0.35 mM (*n* = 5) glucose (Figure 5G, Supplementary Table 8), indicating the possibility of GE-neurons with different glucose sensitivities.

We also determined EC_50_ values separately, for sensitized and desensitized neurons, leading to EC_50_ values of 0.35 ± 0.05 mM (*n* = 3) and 0.85 ± 0.17 mM (*n* = 10; mean ± SE Supplementary Figure 3B, Supplementary Table 8), respectively. All three SN DA neurons with sensitized DA-responses fell into the group of neurons <1 mM, and separate fittings indicated a trend for higher EC_50_ in the desensitized group that was however not significant (Supplementary Figure 3B, Supplementary Table 8).

These data demonstrated fast and reversible glucose-sensing in SN DA neurons, and they indicate two populations of SN DA neurons, according to their glucose-responsiveness: one with an EC_50_ in the euglycemic (0.7–2.5 mM) range of brain glucose-levels, another one in the hypoglycemic range (<0.7 mM) (Routh et al., 2014).

## Discussion

By using an optimized MEA recording and data analysis approach, we analyzed spontaneous pacemaker activities and dopamine-responses of SN neurons in mouse brain slices, along with their modulation by NCS-1 and glucose. Tyrosine-hydroxylase immunostainings of brain slices after recordings allowed to map localizations of individual recording-electrodes within the SN. This approach enabled us to analyze several hundreds of neurons, covering the full SN, without selection bias by the experimenter, and without disturbing the intracellular integrity. Thereby, we identified novel subtypes and physiological functions of SN neurons, in relation to their responses to dopamine, to loss of NCS-1 function, and to changes in extracellular glucose.

### MEA Approaches for Analyzing SN Neurons in Brain Slice Preparations

The principal difference between MEA and patch clamp recordings is that either extracellular spikes or intracellular action potentials are recorded. The major advantage of the MEA approach is its relatively easy and fast execution, and the recording of multiple neurons in parallel, compared to perforated patch clamp or on-cell recordings (Spira and Hai, 2013). MEA allows a more unbiased analysis of all cellular populations in a region of interest, as choosing the cells to analyze by the experimenter is omitted. However, analysis of data from MEA recordings is more complicated compared to patch clamp recordings (Negri et al., 2020). This is due to the sheer size of data received from MEA recordings that needs to be further processed for automated spike detection and further downstream-analysis (Stevenson and Kording, 2011; Rey et al., 2015; Mahmud and Vassanelli, 2016, 2019). The file-size from our raw unprocessed MEA recordings was up to ~16 GB, depending on the length of the recordings. A crucial issue is to extract those signals that reflect cell-derived single unit activities (SUAs), while avoiding analysis of the same cell recorded from more than one electrode. The fact that SN DA neurons display *in vitro*, even in full synaptic isolation, a regular low-frequency pacemaker activity (~0.5–3 Hz with a maximal firing rate ~10 Hz) and broad action potentials (>2 ms) (Wolfart and Roeper, 2002; Lammel et al., 2008; Khaliq and Bean, 2010; Poetschke et al., 2015) facilitates their identification. However, pacemaker-activity can be less regular, due to physiological variation (Marinelli and McCutcheon, 2014), and in particular in MEA recordings as spike detection can fail to recognize all spikes belonging to a single neuron. A disadvantage of MEA approaches is that it is not exactly clear from which cell a recording is derived, but only the placement/coordinates of the respective electrode that recorded its activity (Obien et al., 2019). Hence, the maps given here (Figure 2) reflect the location of the electrodes that recorded the respective SUAs with the responses, as indicated. We cannot rule out that our collectives of analyzed SN cells do also contain a fraction of VTA neurons and/or VTA-like calbindin-positive dorsal tier SN DA neurons, as their locations and their *in vitro* electrophysiological properties are partly overlapping (Margolis et al., 2006; Lammel et al., 2008; Khaliq and Bean, 2010; Krashia et al., 2017).

The number of neurons we recorded with our MEA approach are comparable to other MEA brain slice studies, e.g., in the hypothalamus (Hanna et al., 2019). Our general findings in respect to the dopamine-response are in line with other studies of SN neurons in horizontal brain slices from ~1 month old rats (Geracitano et al., 2005; Berretta et al., 2010), and with a recent MEA study, where cultured DA neurons were analyzed, derived from dissected embryonal (E15) TH-GFP mouse brains, expressing the enhanced green fluorescent protein (GFP) under the tyrosine-hydroxylase promoter (Tomagra et al., 2019). In the latter study, micro-graphitic single crystal diamond Multi Electrode Arrays (μG-SCD-MEAs) were used, that allow, in addition to recording of electrical activity, amperometric analysis of dopamine release. We utilized 3D tip-shaped electrodes that entered the tissue and thus got closer to the neurons in the slice (Heuschkel et al., 2002).

### Dopamine-Excited Cells in the SN

While with our MEA recordings we detected a large population of SN DA neurons that increased their electrical activity in response to dopamine, we never saw this type of response in our datasets of perforated patch clamp and on-cell recordings. However, pacemaker activities and dopamine-responses of the dopamine-inhibited SN neurons, recorded with perforated patch clamp recordings were not significantly different from those recorded with our MEA approach (Figure 1G, Supplementary Table 2), and (Liss et al., 2005; Lammel et al., 2008; Dragicevic et al., 2014; Poetschke et al., 2015). To our knowledge, dopamine-excited neurons have also not been reported in other brain slice patch clamp studies. Possible explanation might be e.g., a preferential selection by the experimenter or differential accessibility of subpopulations of SN DA neurons with patch clamp approaches, or methodological impacts of either approach.

DA-excited neurons were also described in a similar brain slice MEA study, analyzing rat SN DA neurons in horizontal brain slices in response to 30 μM dopamine, applied for 1–2 min (Berretta et al., 2010), and in the MEA study analyzing cultured, embryonal, TH-GFP mice derived neurons (Tomagra et al., 2019). In the latter, L-DOPA (20 μM), the precursor of dopamine, was given, and it also either excited or inhibited DA neurons. Further, they found TH-GFP positive neurons that were excited by dopamine with MEA as well as with patch clamp whole-cell recordings (personal communication, Valentina Carabelli). However, in another MEA brain slice study of TH-GFP mice (PN17–PN30), almost all SN neurons in horizontal slices were inhibited by dopamine (30 μM for 2-3 min), dopamine-excited cells were not reported (Krashia et al., 2017). The proportion of dopamine (or L-DOPA)—inhibited and—excited SN neurons was about 50:50% in our study, 81:2% (remaining 17% were insensitive) and 80:17% (remaining 3% were insensitive) in the other MEA slice, and MEA DA culture study, respectively (Berretta et al., 2010; Tomagra et al., 2019). Discrepancies in the detection of dopamine-excited SN cells are likely caused by different ages and/or types of analyzed neurons, or technical differences.

What is the anatomical and neurochemical nature of the dopamine excited SN cells? By mapping the electrodes that recorded DA-excited cells, we found no specific anatomical localization. With our approach, we could neither identify the projections of DA-excited cells, nor whether they were dopaminergic or non-dopaminergic—or even neurons. However, the L-DOPA/dopamine excited SN cells in Tomagra et al. (2019) displayed a positive fluorescence TH-GFP signal, indicating these cells were indeed dopaminergic neurons—or at least tyrosine-hydroxylase positive cells. Together with the high number of dopamine-excited SN cells detected, we conclude that they are dopaminergic, and a subpopulation of SN DA neurons exists that is excited by dopamine. The mechanism for the dopamine-mediated stimulation of presumed SN DA neurons is unclear. Several ways are possible. It could be mediated directly, via stimulatory D1-type dopamine receptors [a small population of D1/D5 expressing SN DA neurons is described (Liss and Roeper, 2004; Hetzel, 2008; Jang et al., 2011)]. Or D2-AR could stimulate depolarizing low threshold T-Type Cav channels, as described for calbindin-negative ventral tier SN DA neurons (Evans et al., 2017). Also, depolarization by the electrogenic dopamine-transporter (DAT, co-importing netto one positive charge with each dopamine molecule), in the absence or full desensitization of inhibitory D2-AR, offers a possible mechanism (Sonders et al., 1997; Ingram et al., 2002; Carvelli et al., 2004). Or stimulation could be mediated more indirectly, e.g. by D2-receptor inhibition of inhibitory interneurons that control the activity of DA neurons, as described for VTA DA neurons (Nestler, 2005; Morales and Margolis, 2017; Bouarab et al., 2019). Identity, mechanisms, and functions of DA-excited neurons will be addressed in future studies.

### Two Types of Dopamine Inhibited SN DA Neurons: Loss of NCS-1 Function and Elevated Glucose Promote Desensitized D2-AR Response

We identified SN DA neurons with either sustained or desensitized dopamine inhibition of spontaneous activity. As MEA allowed us to record much more neurons, compared to our previous patch clamp studies, n-numbers were high enough to allow separate analysis of neurons with sensitized and desensitized D2-AR responses as two populations. This analysis revealed that time course and degree of desensitization were surprisingly very similar in all analyzed neurons with desensitized responses, in all conditions. Hence, SN DA neurons do not display a spectrum of autoreceptor responses with different degrees of desensitization, as previously assumed (Dragicevic et al., 2014; Poetschke et al., 2015), but only two states of D2-AR responses: sensitized or desensitized. The dopamine-responses when NCS-1 function was lost and when extracellular glucose was elevated support this binary view, as again, the kinetics of individual desensitization was not altered, but only the number of SN DA neurons with desensitized dopamine responses. We propose that SN DA neurons switch between these two distinct states, rather than gradually change desensitization of D2-AR responses.

Accordingly, we conclude that the previously reported less pronounced desensitization of dopamine responses—in adult vs. juvenile mice, in cocaine vs. saline treated mice, in Cav1.3 KO vs. wildtype mice (Dragicevic et al., 2014; Poetschke et al., 2015; Robinson et al., 2017b)—identified by analysis of SN DA neurons as one population, is reflecting a reduction of the number of SN DA neurons with desensitized responses, not a change in desensitization-kinetics, similar as reported here for loss of NCS-1 function and elevated glucose. An alternative explanation could be that due to NCS-1 function or at lower glucose levels SN DA neurons with desensitized D2-AR responses die, or were no longer recorded by MEA, and thus we detect more neurons with sustained dopamine responses. However, our very long and metabolically stressful glucose experiments for dose-response curves that ended with dopamine-application and still showing ~75% desensitized responses, argue against this.

We have not yet addressed the mechanism of the proposed switch from desensitized to sensitized D2-AR responses. Mechanisms of D2-receptor desensitization have been identified mainly in heterologous systems. Best described is receptor-phosphorylation and subsequent beta-arrestin mediated internalization (Beaulieu et al., 2015; Chen et al., 2020). However, for D2-ARs, an internalization-independent, Ca^2+^ involving desensitization mechanism is described (Gantz et al., 2015; Robinson et al., 2017a). Our data would support the latter mechanism. The electrogenic DAT (importing 2 Na^+^ and 1 Cl^−^ ion with each dopamine molecule), could also be involved in mediating desensitization of inhibitory dopamine responses in SN DA neurons, possibly in interplay with D2-receptors and other conductances, as already described (Sonders et al., 1997; Ingram et al., 2002; Carvelli et al., 2004; Aversa et al., 2018).

What is the physiological function of altered somatodendritic D2-AR responses? The physiological function of somatodendritic local dopamine-release in general is still unclear. However, it creates activity-related increases in extracellular dopamine in the SN that inhibit autoactivity and the activity of neighboring neurons, and could contribute to synchronization (Joshua et al., 2009; van der Velden et al., 2020). D2-AR function reduces SN DA neuron activity, and thus also excitotoxicity related processes (Rice and Patel, 2015; Duda et al., 2016). A lower number of SN DA neurons with desensitized D2-AR response would prolong these effects of dopamine, and thus reduce the overall-activity of SN DA neurons and related metabolic stress. Accordingly, SN DA neurons from NCS-1 KO mice with less sensitized SN DA responses should be more vulnerable to degenerative stressors—and this is indeed the case in a PD model (Benkert et al., 2019; Simons et al., 2019).

In this context, it is noteworthy that in general, the number of SN DA neurons with desensitized inhibitory dopamine responses appeared to be decreased when the brain slices were not in optimal condition, e.g., due to suboptimal slice-preparation, mounting, or perfusion. D2-AR responses of SN DA neurons from adult mice were particularly sensitive to these kinds of stressors.

### SN DA Neurons Are Glucose Sensors (GE-Neurons)

SN DA neurons are particularly vulnerable to metabolic stressors (Dragicevic et al., 2015), express insulin receptors, and are regulated by insulin (Figlewicz et al., 2003; Fiory et al., 2019), but physiological glucose sensing had not yet been reported. We show here for the first time that most SN DA neurons (75%) display glucose sensing properties, with higher pacemaker-activities at higher glucose levels (as defined for glucose-excited GE-neurons), independently from their D2-AR responses. GE-neurons are typically found in the hypothalamic and brainstem nuclei (Fioramonti et al., 2017; Guemes and Georgiou, 2018; Lopez-Gambero et al., 2019). We demonstrated here in individual SN DA neurons a fast and reversible decrease of activity with lower glucose, and an increase in response to higher glucose, with EC_50_ values in the (patho-) physiological range of brain glucose levels. Individual dose-response curves indicate that SN DA neurons might display different glucose sensitivities, with one population displaying EC_50_ values in the euglycemic range (EC_50_: ~2.0 mM) and another one within hypoglycaemic (EC_50_: ~0.35 mM) brain glucose levels. These values correspond well with those described for GE neurons in the lateral hypothalamus, with an EC_50_ of ~0.8 mM glucose (Burdakov et al., 2005) and in the nucleus arcuatus, with ~2 mM (Wang et al., 2004).

What is the physiological function of glucose sensing in SN DA neurons? Brain glucose-levels are lower compared to blood glucose levels (~2 mM and 7–8 mM, respectively; Routh et al., 2014; Fioramonti et al., 2017). Glucose is the primary fuel for the brain, and its levels remain relatively constant, even when plasma levels are fluctuating (Dunn-Meynell et al., 2009; Hwang et al., 2019). However, brain glucose levels can reach locally much higher levels, and corresponding so-called high-GE-neurons are described, sensing glucose above 5 mM, up to 10, or even over 20 mM (Routh, 2010; Fioramonti et al., 2017; Alvarsson and Stanley, 2018), reflected by our chosen glucose concentrations (ranging from 0.5 to 25 mM).

Reduced activity at lower glucose levels will reduce the activity of the Na^+^/K^+^ ATPase, and thus ATP consumption and metabolic stress in metabolic demand. Similar as it has been described for SN DA neurons in horizontal brain slices from juvenile rats, where glucose levels were drastically reduced from 10 mM down to zero mM (Marinelli et al., 2000, 2001). But what could be long term consequences? A number of studies indicate that activity of SN DA neurons is not only crucial for dopamine release and related functions, but also for their maintenance and survival, in line with the classical “use it or lose it” principle of neuronal loss (reviewed e.g., in Swaab et al., 2002; Duda et al., 2016; Michel et al., 2016). Thus, while reduced activity of SN DA neurons at low glucose levels likely has acute beneficial effects, in the long run it could contribute to degeneration and PD, similar as shown for K-ATP channel activity (Liss et al., 2005; Schiemann et al., 2012; Duda et al., 2016).

The molecular mechanisms of glucose sensing in SN DA neurons is currently unclear. However, for GE- and GI-neurons in other brain regions mechanisms have been identified, involving plasmalemma glucose transporters for glucose uptake (GLUT1-14, SGLT1-6), hexokinases/glucokinase (GK) for intracellular glucose phosphorylation, and a variety of downstream ion channels and receptors (reviewed e.g., in Routh et al., 2014; Fioramonti et al., 2017; Lopez-Gambero et al., 2019; Stanley et al., 2019; Hirschberg et al., 2020). GE-neuron glucose sensing is best described in neurons of the ventromedial hypothalamus (VMH), and it is similar to the mechanism in pancreatic beta cells, involving GK, GLUT2, KATP, and Cav channels (Pozo and Claret, 2018; Rorsman and Ashcroft, 2018), all of them expressed in SN DA neurons. Other proteins involved in GE-neuron glucose sensing are metabolically sensitive chloride channels, purinergic receptors, sweet taste receptors (T1R2/3) and transient receptor potential channels (TRPC3, TRPM). For GI-neuron glucose sensing mechanisms, in particular the Na^+^/K^+^ ATPase, as well as AMP Kinase, nNOS/nitric oxide, CFTR chloride channels, glutamate receptors, or two-pore-domain potassium channels (K2P, TASK) have been described (Marinelli et al., 2000, 2001) Kv7 potassium channels and volume regulated anion channels (VRAC) have also been linked to glucose sensing (Stuhlmann et al., 2018; Di Fulvio and Aguilar-Bryan, 2019; Manville and Abbott, 2020).

Future studies will address the molecular mechanisms of glucose sensing in SN DA neurons. Our first experiments indicate a complex mechanism that is not simply explained by activation of K-ATP channels or A-type K^+^ channels, as described for pathophysiological reduction of SN DA activity in response to PD stressors (Liss et al., 1999, 2005; Subramaniam et al., 2014; Dragicevic et al., 2015; Subramaniam and Roeper, 2017). Dissecting these mechanism is particularly relevant in view of the high vulnerability of SN DA neurons in PD, and the emerging link between pancreatic beta cells and SN DA neurons, as well as between type II diabetes (T2DM) and PD (Eberhard, 2013; Hassan et al., 2020; Sportelli et al., 2020). T2DM, with elevated blood glucose levels and increasing insulin resistance, seemingly increases the risk for developing PD later in life, and both diseases share similar pathomechanisms (Camargo Maluf et al., 2019; Cheong et al., 2020). Understanding physiological and pathophysiological glucose sensing of SN DA neurons, also in context of T2DM, will lead to a better understanding of the high vulnerability of these neurons to degeneration in PD. It could help identifying early changes in SN DA neurons, before they degenerate, and thus help identifying PD patients before motor systems manifest and most SN DA neurons are already lost—a prerequisite for neuroprotective PD therapies.

## Data Availability Statement

The original contributions presented in the study are included in the article/Supplementary Material, further inquiries can be directed to the corresponding author/s.

## Ethics Statement

The animal study was reviewed and approved by the Regierungspräsidium, Referat 35, Konrad Adenauer Strasse 20, 72072 Tübingen.

## Author Contributions

NM and KK performed MEA experiments and data analysis. CP carried out perforated patch clamp experiments. NM and KK performed immunohistology. MF and AD carried out initial MEA pilot-experiments. BL designed the study and data analysis. BL and NM wrote the manuscript. All authors revised the manuscript.

## Conflict of Interest

The authors declare that the research was conducted in the absence of any commercial or financial relationships that could be construed as a potential conflict of interest.

## Abbreviations

ACSF: artificial cerebrospinal fluid
AMP: adenosine monophosphate
Arc: arcuate nucleus
Cav: voltage-gated calcium channel
CFTR: cystic fibrosis transmembrane conductance regulator
CV: coefficient of variation
D2-AR: autoreceptor of the D2 type
DA: dopamine
DMN: dorsomedial nuclei
DMV: dorsal motor nucleus of the vagus
DNIP: D2/NCS-1 interaction preventing peptide
EC50: half maximal effective concentration
GE: glucose excited
GFP: green fluorescent protein
GI: glucose inhibited
GIRK: G-protein coupled inwardly rectifying K^+^
GK: glucokinase
GLUT: glucose transporter
HT: hypothalamus
ISI: interspike interval
K2P (TASK): two-pore domain potassium
K-ATP: ATP-sensitive K^+^
KO: knockout
K_v_: voltage-gated potassium channel
L-Dopa: Levodopa
LH: lateral hypothalamus
MEA: multi-electrode array
MUA: multi unit activity
NCS-1: neuronal Ca^2+^ sensor
PBM: parabrachial medial nucleus
PBS: phosphate buffered saline
PCA: principal component analysis
PD: Parkinson's disease
PN: post-natal
PVN: periventricular nucleus
SD: standard deviation
SE: standard error
SEM: standard error of the mean
SGLT: sodium glucose co-transporter
SN: substantia nigra
srDNIP: scrambled DNIP
SUA: single unit activity
T1R: sweet taste receptor
T2DM: diabetes mellitus type 2
TH: tyrosine hydroxylase
TRPC: transient receptor potential channel
VMH: ventromedial hypothalamus
VMN: ventromedial nucleus
VRAC: volume regulated anion channels
VTA: ventral tegmental area
WT: wildtype.
