# Supplementary material for: Multi-Electrode Array Analysis Identifies Complex Dopamine Responses and Glucose Sensing Properties of Substantia Nigra Neurons in Mouse Brain Slices
Source: Front Synaptic Neurosci. 2021 Feb 26;13:635050. doi: 10.3389/fnsyn.2021.635050 (PMC7952765; doi:10.3389/fnsyn.2021.635050)
Supplement: Supplementary file 1 [file Data_Sheet_1.pdf]

Supplementary Material

1 Supplementary Figures

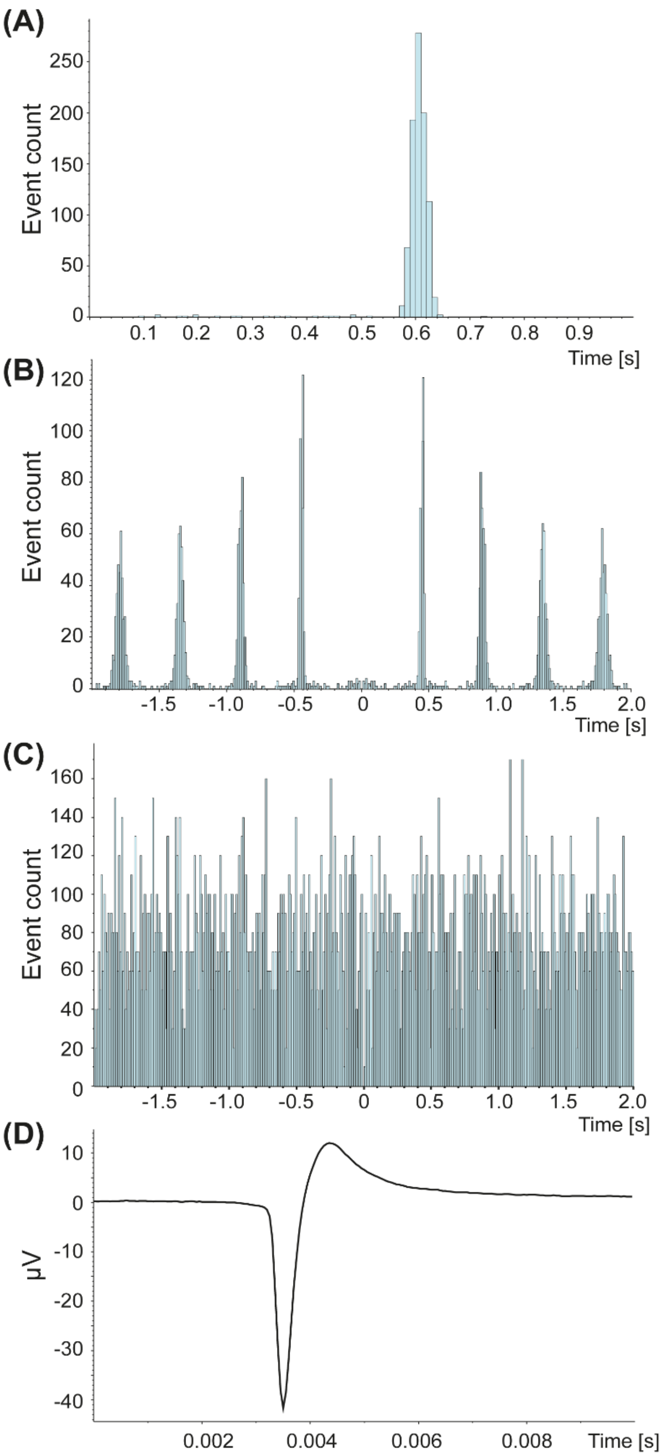

Figure S1: Example of a MEA recorded unit, fulfilling preselection criteria for SUA, derived from an SN DA neuron. Experiments, MEA recordings, data analysis and presentation as in Figure 1. Analysis (carried out with Spike2) of an identified single unit activity (SUA), over a ten minute control period at the beginning of the recording. **(A)** Interval histogram (width: 1; bin size: 0.01) of sorted events (i.e. action potentials), showing a lack of events during the first 200 ms, and one clear, large peak of events, defined as the number of spikes occurring at the same timepoint. **(B)** Auto-correlogram, plotting the event count of one unit against lag (sec., width: 4; bin size: 0.01; offset: 2). Note the clear and regular activity. **(C)** Cross-correlation histogram between two units, recorded by the same electrode (settings as in B). Event count plotted against lag. Note a lack of events at zero (defining the lack of simultaneously occurring spikes). The irregular distribution of events implies the independence of the two units. **(D)** Averaged event of a SUA, displaying the typical shape of an extracellularly recorded action potential of a SN DA neuron (width 0.01; offset: 0).

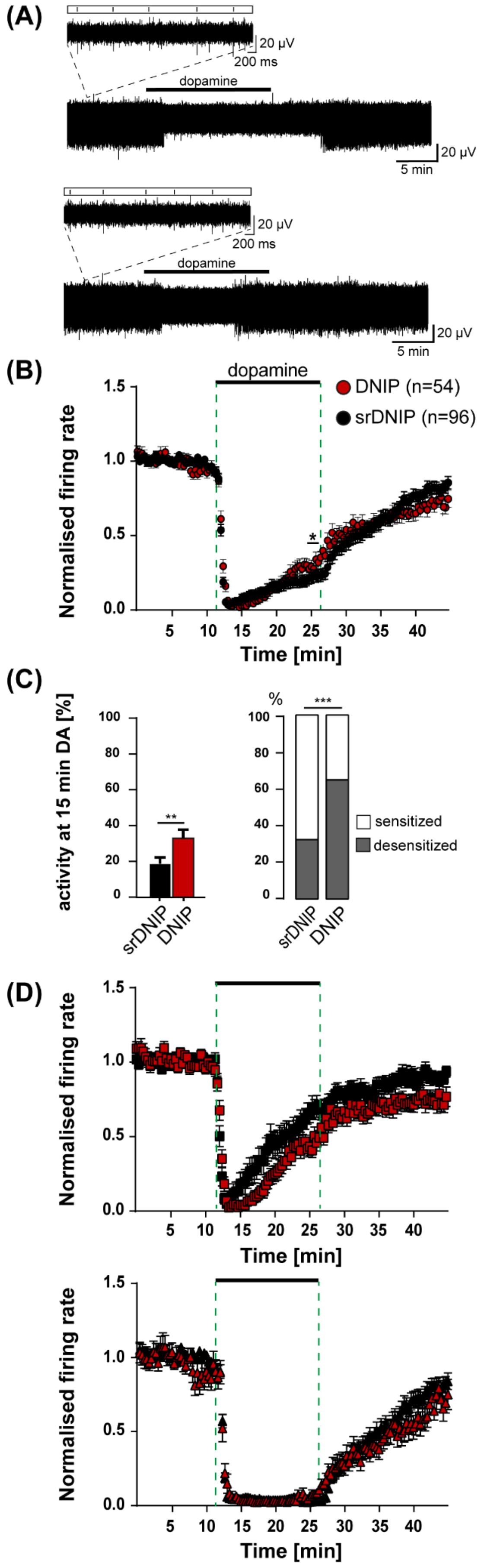

(A)
20 µV
200 ms
dopamine
20 µV
5 min
20 µV
200 ms
dopamine
20 µV
5 min
(B)
dopamine
DNIP (n=54)
srDNIP (n=96)
Normalised firing rate
Time [min]
(C)
activity at 15 min DA [%]
srDNIP
DNIP
%
sensitized
desensitized
(D)
Normalised firing rate
Time [min]

Figure S2: Prevention of NCS-1 binding to D2-autoreceptors increases the number of SN DA neurons with desensitized inhibitory dopamine-responses. Experiments, MEA recordings, data analysis and presentation as in Figure 3. Data were derived from adult C57BL/6 mice. Brain slices were preincubated either in srDNIP (n=96; scrambled D2/NCS-1 interacting prevention peptide), or DNIP (n=54), and recorded in ACSF containing 2.5 mM glucose. **(A)** Exemplary traces of a SN DA neuron with sensitized (upper) and with desensitized (lower) inhibitory dopamine responses (mediated by D2-autoreceptors, D2-AR). Bath application of 100 µM dopamine indicated by horizontal bars. Inserts display enlargements of two seconds. **(B)** Normalized mean firing rates plotted over time for all analysed SN DA neurons, in the presence of either srDNIP or DNIP. **(C)** Mean relative firing frequencies in the last minute of dopamine (left), and percentages of cells with desensitized dopamine-responses (grey, right), for SN DA neurons, in the presence of 10 µM DNIP, or 10 µM srDNIP. **(D)** Data from (B), plotted separately for SN neurons with desensitized (upper, srDNIP: n=31, DNIP: n=37) and with sensitized inhibitory dopamine responses (lower, srDNIP: n=65, DNIP: n=17). Data are given as mean ± SEM, Significances/p-values according to two-way ANOVA with Sidak´s multiple comparison post-hoc test, Chi-square, and unpaired Mann-Whitney-test. All data and statistics are detailed in Tables S3&4.

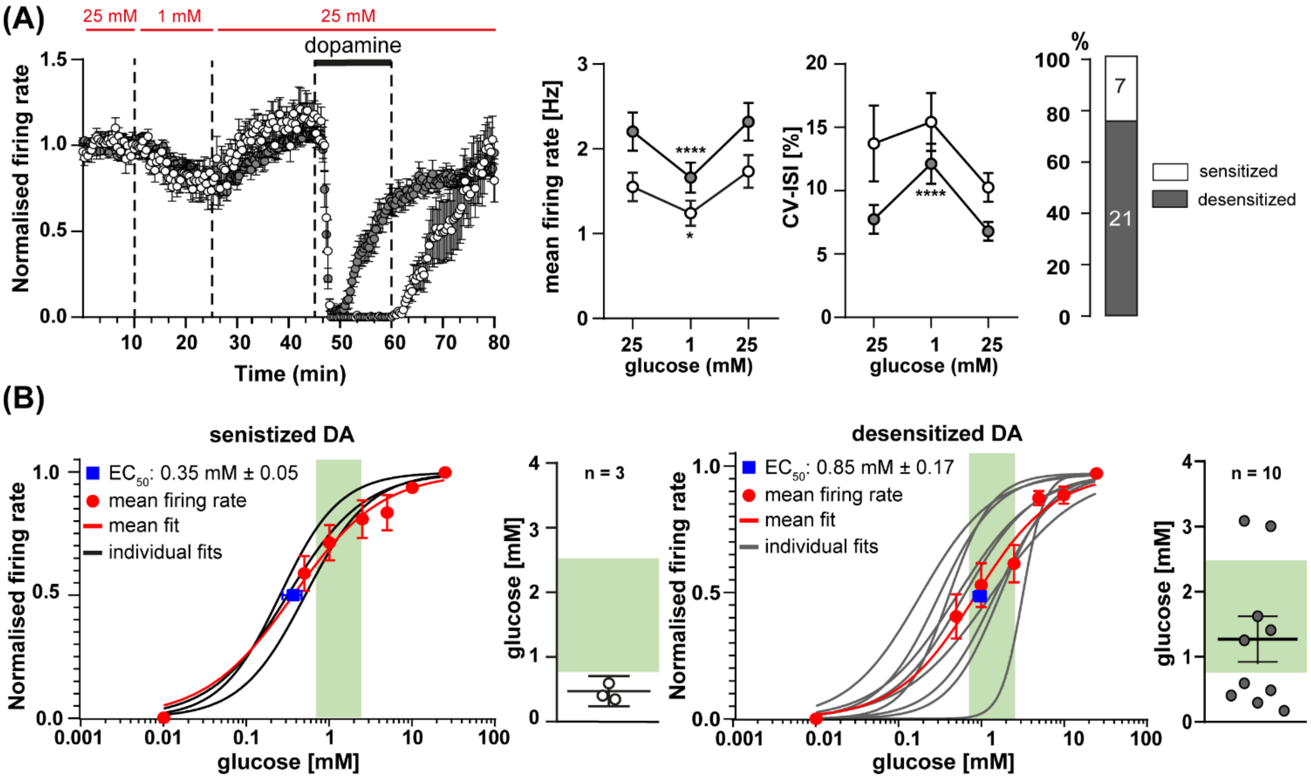

Figure S3: Glucose sensitivity of SN DA neurons does not correspond with inhibitory dopamine response types. Experiments, MEA recordings, data analysis and presentation similar as in Figure 5. Data from Figure 5 are plotted here separately for SN DA neurons with either desensitized (grey) or sensitized (white) inhibitory dopamine responses. **(A) Left panel:** Normalized mean firing rates of all analyzed SN DA neurons are plotted over time (desensitized: n=21, sensitized: n=7). Bath application of different glucose concentration (red), and of 100 µM dopamine (black) indicated by horizontal bars. **Middle panels**: Mean non-normalized firing rates and pacemaker precision (given as CV-ISI) during first ten minutes of the recording in 25 mM glucose, in 1mM (last ten minutes), and back in 25 mM glucose (last ten minutes), for SN DA neurons with either desensitized (grey) or sensitized (white) inhibitory dopamine responses. **Right panel:** Number / percentage of SN DA neurons from Figure 5 with desensitized D2-AR responses (grey). **(B)** Data from Figure 5 E/F, plotted separately for SN DA neurons with either desensitized (left panels, n=3) or sensitized (right panels, n=10) inhibitory dopamine responses. EC_{50} values were derived from fitting (according to the Hill-equation) the mean fit of the fits for each individual neuron (red). Data is given as mean ± SE. The range of physiological brain glucose levels is indicated in green, [0.7-2.5 mM], according to Routh et al. 2014. Data are given as mean ± SEM. Significances/p-values according to paired Friedman-test with Dunn´s multiple comparison post-hoc test. All data and statistics are detailed in Table S7&S8.

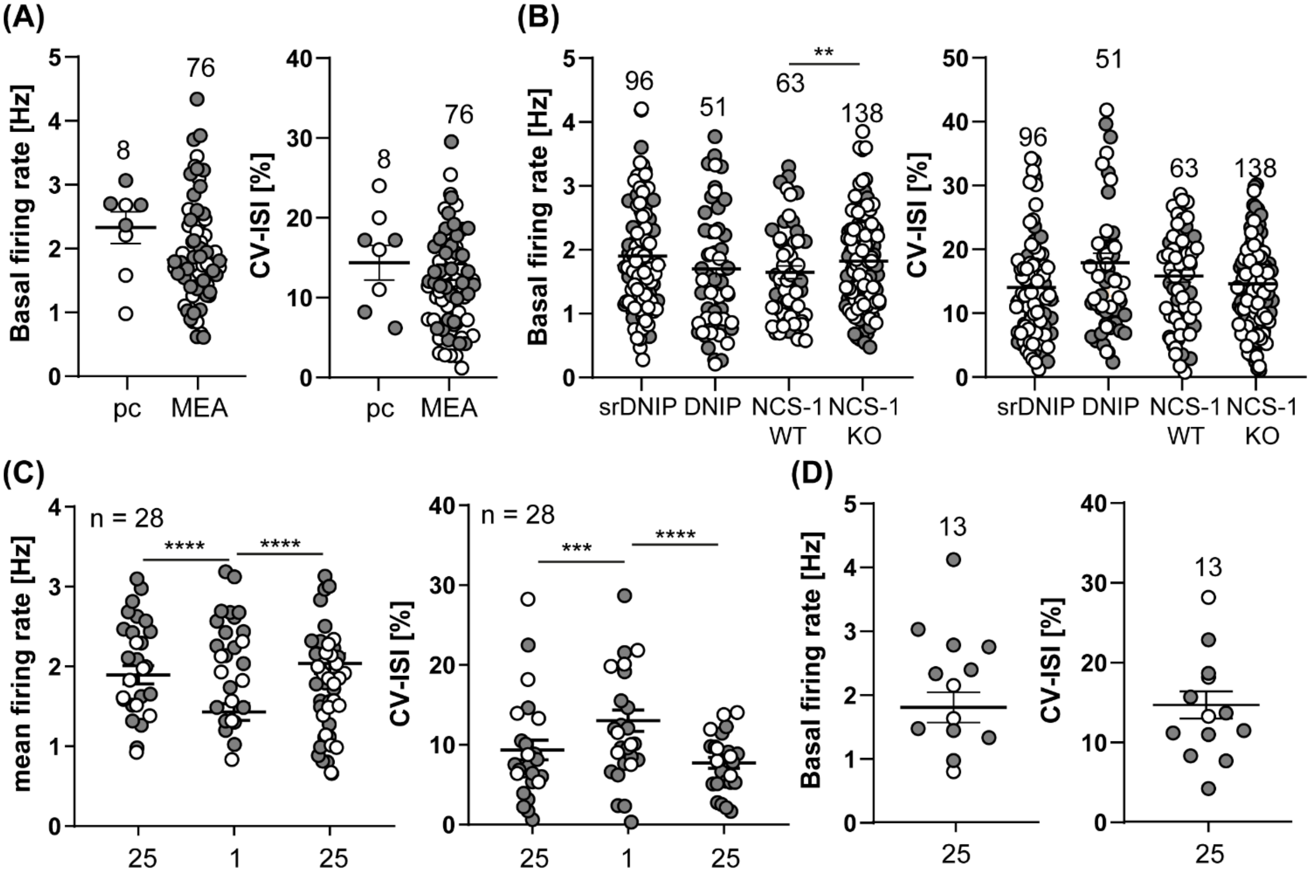

Figure S4: Basic pacemaker activities of SN DA neurons, displayed in Figures 1G, 3, 4, and 5. Pacemaker frequencies are given as basal firing rates of the first 10 minutes of recording, or mean firing rates (D). Pacemaker precision is given as coefficient of variance of the interspike interval (CV-ISI). SN DA neurons with desensitized dopamine D2-AR responses are plotted in grey, those with sensitized responses in white. **(A)** Data for all recorded SN DA neurons shown in Figure 1G. **(B)** Data for all recorded SN DA neurons shown in Figure 3&S2. **(C)** Data for all recorded SN DA neurons shown in Figure 5A-C. **(D)** Data for all recorded SN DA neurons shown in Figure 5E-G. Data are given as mean ± SEM. Significances/p-values according to unpaired Mann-Whitney-test, unpaired Kruskal-Wallis-test or paired Friedman test with Dunn´s multiple comparison post-hoc test. All data and statistics are detailed in Table S2, S3, S5, S7&S8.

2 Supplementary tables

Table S1: Comparison of pacemaker activities and dopamine responses of SN cells, recorded with MEA-technique. Data from experiment shown in Figure 1F/G and Figure 2 (in 2.5 mM glucose). Pacemaker frequencies are given as basal firing rate (mean firing rate during the first 10 minutes of recordings). Pacemaker precision is displayed as % coefficient of variance of the interspike interval (CV-ISI). Mean firing frequencies in the last minute of dopamine are given as "activity at min 15 in DA [Hz]". DA-excited "silent" neurons only displayed activity in dopamine, but not before or after wash-out (D2-AR dopamine autoreceptor of the D2-Type). n gives the number of analyzed cells (in brackets, the relative % of this cell-type is given), N gives the number of analyzed mice. Data are given as mean ± SD. Data sets marked by (#) were not normally distributed. Significances/p-values according to unpaired Kruskal-Wallis-test with Dunn´s multiple comparison post-hoc test. P-values indicating significant differences are given in bold red.

**Dopamine response**	**Juvenile C57BL/6**				
	**Basal firing rate [Hz]**	**CV-ISI [%]**	**Activity at min 15 in DA [Hz]**	**n**	**N**
DA-excited, "silent", no spontaneous activity	0	-	3.39±0.97	26 (38%)	7
DA-excited, spontaneous activity	2.45±1.4	12.22±8.79	5.21±2.1	4 (6%)	7
DA-inhibited, sensitized D2-AR	1.44±0.56	12.62±8.2 #	0.07±0.26 #	21 (31%)	7
DA-inhibited, desensitized D2-AR	1.69±0.64	11.21±6.89	0.76±0.55	17 (25%)	7
p-values	0.16	0.884	excited: 0.425<br>inhibited: **<0.0001**<br>all: <0.0001		
**Dopamine response**	**Adult C57BL/6**				
	**Basal firing rate [Hz]**	**CV-ISI [%]**	**Activity at min 15 in DA [Hz]**	**n**	**N**
DA-excited, "silent", no spontaneous activity	0	-	4.2±2.6 #	22 (22%)	7
DA-excited, spontaneous activity	3.21±2.99 #	16.07±10.48 #	8.8±6.7 #	20 (20%)	7
DA-inhibited, sensitized D2-AR	2.1±0.79 #	8.78±5.23 #	0.01± 0.03 #	30 (30%)	7
DA-inhibited, desensitized D2-AR	2.33±0.77	10.69 ± 5.22	1.74± 1.7 #	28 (28%)	7
p-values	0.496	inhibited sens. vs. excited: **0.02**	excited: **0.015**<br>inhibited: **<0.0001**<br>all: **<0.0001**		

Table S2: Comparison of pacemaker (frequencies and precision), and dopamine responses of SN DA neurons, recorded with MEA or perforated patch-clamp techniques. Data from experiment shown in Figure 1G (in 2.5 mM glucose). Pacemaker frequencies are given as basal firing rate (mean firing rate during the first 10 minutes of recordings). Pacemaker precision is displayed as coefficient of variance of the interspike interval (CV-ISI). Mean relative activities in the last minute of dopamine are given as "activity at min 15 in dopamine [%]". DA-excited "silent" neurons only displayed activity in dopamine, but not before or after wash-out. n gives the number of analyzed cells (in brackets, the relative % of neurons with desensitized inhibitory dopamine-responses is given), N gives the number of analyzed mice. Data are given as mean ± SEM. Data sets marked by (#) were not normally distributed. Significances/p-values according to unpaired Mann-Whitney-test and Chi-square test.

**Recording-paradigm** (data sets from Fig. 1G)	**Basal firing rate [Hz]**	**CV-ISI [%]**	**Activity at min 15 in DA [%]**	**n**	**N**
MEA-recordings	1.9±0.78 #	11.72±5.8	25±33.68 #	76 (44%)	7
Perforated patch-clamp recordings	2.3±0.7	15±6.1	24±30.4 #	8 (50%)	3
p-values	0.123	0.166	0.591	0.5757	

Table S3: Comparison of pacemaker (frequencies and precision), and dopamine responses of SN DA neurons. Data from experiments shown in Figure 3 and S2, derived from adult NCS-1 KO, NCS-1 WT, and from C57BL/6 mice in 10 µM DNIP or srDNIP (D2/NCS-1 interacting prevention peptide), in 2.5 mM glucose. Pacemaker frequencies are given as basal firing rate (mean firing rate during the first 10 minutes of recordings). Pacemaker precision is displayed as coefficient of variance of the interspike interval (CV-ISI). Mean relative activities in the last minute of dopamine are given as "activity at min 15 in DA [%]". "Pooled": all dopamine-inhibited neurons were analyzed as one population. "Desens": only SN DA neurons with desensitized inhibitory dopamine responses were analyzed. "Sens": only SN DA neurons with sensitized (sustained) inhibitory dopamine responses were analyzed. n gives the number of analyzed cells; N gives the number of analyzed mice. Data sets marked by (#) were not normally distributed. Significances/p-values according to unpaired Kruskal-Wallis test with Dunn´s multiple comparison post-hoc test. P-values indicating significant differences are given in bold red.

		NCS1-KO						NCS-1 WT §							
		**Mean**	**SEM**	**SD**	**Median**	**95% CI**	**n/N**		**Mean**	**SEM**	**SD**	**Median**	**95% CI**	**n/N**	**p-value**
**Basal firing rate [Hz]**	Pooled #	1.79	0.06	0.70	1.73	1.7-1.9	138/6	Pooled #	1.61	0.09	0.79	1.46	1.4-1.8	63/6	**0.0416**
	Desens	1.67	0.07	0.66	1.63	1.5-1.8	77/6	Desens	1.93	0.17	0.78	1.91	1.6-2.3	22/6	0.157
	Sens	1.95	0.09	0.73	1.88	1.8-2.1	61/6	Sens #	1.45	0.11	0.75	1.21	1.2-1.7	41/6	**<0.0001**
**CV ISI [%]**	Pooled	13.80	0.55	6.44	13.76	12.7-14.9	138/6	Pooled	15.01	0.96	7.59	15.01	13.1-16.9	63/6	0.2623
	Desens	14.79	0.76	6.65	14.68	13.3-16.3	77/6	Desens	13.72	1.51	7.10	13.54	10.6-16.9	22/6	0.5839
	Sens	12.43	0.76	5.92	13.19	10.9-13.9	61/6	Sens	15.64	1.20	7.73	17.46	13.2-18.1	41/6	**0.0218**
**Activity at min 15 in DA [%]**	Pooled #	41.95	4.12	48.43	34.26	13.2-27.7	138/6	Pooled #	21.34	4.40	34.90	0	12.6-20.3	63/6	**0.008**
	Desens #	75.08	4.72	41.38	69.79	65.7-84.5	77/6	Desens #	61.11	6.97	32.70	70.76	46.6-75.6	22/6	0.559
	Sens #	0.13	0.09	0.67	0	0.04-0.3	61/6	Sens #	0.14	0.09	0.56	0	0.04-0.3	41/6	0.382
		**DNIP**						**srDNIP**							
		**Mean**	**SEM**	**SD**	**Median**	**95% CI**	**n/N**		**Mean**	**SEM**	**SD**	**Median**	**95% CI**	**n/N**	**p-value**
**Basal firing rate [Hz]**	Pooled #	1.67	0.13	0.95	1.53	1.4-1.9	54/5	Pooled #	1.87	0.09	0.83	1.71	1.7-2.0	96/5	0.1378
	Desens	1.80	0.17	0.98	1.72	1.5-2.1	37/5	Desens	2.05	0.16	0.87	1.96	1.7-2.4	65/5	0.2754
	Sens #	1.32	0.19	0.82	0.93	0.9-1.7	17/5	Sens #	1.76	0.10	0.82	1.67	1.6-1.9	31/5	**0.0213**
**CV ISI [%]**	Pooled #	17.10	1.50	10.68	14.99	14.0-20.1	54/5	Pooled #	13.22	0.78	7.56	12.28	11.7-14.8	96/5	0.0547
	Desens #	16.62	2.05	12.15	11.61	12.1-20.4	37/5	Desens	11.91	1.18	6.57	11.26	9.5-14.3	65/5	0.4626
	Sens	19.13	2.27	9.90	17.33	14.5-23.9	17/5	Sens #	15.27	1.40	11.28	13.11	12.5-18.1	31/5	**0.0341**
**Activity at min 15 in DA [%]**	Pooled #	41.25	7.63	56.03	29.37	26.0-56.5	54/5	Pooled #	20.42	3.67	35.91	0	13.2-27.7	96/5	**0.0015**
	Desens #	58.21	10.15	60.05	52.51	37.6-78.8	37/5	Desens #	62.59	6.64	36.99	55.14	49.0-76.2	65/5	0.187
	Sens #	1.19	0.40	1.66	0.09	0.3-2.0	17/5	Sens #	0.31	0.13	1.06	0	0.05-0.6	31/5	0.656

§: WT data were pooled as NCS-1+/+ littermate data were not significantly different to that from non-littermate NCS-1+/+, or from C57BL/6J mice.

Table S4: Number and ratio of SN DA neurons with sensitized and desensitized inhibitory dopamine responses. Data from experiment shown in Figures 3, S2, and Table S3, derived from adult NCS-1 KO, NCS-1 WT, and from C57BL/6 mice in 10 µM DNIP or srDNIP (D2/NCS-1 interacting prevention peptide), in 2.5 mM glucose, as indicated (D2-AR dopamine autoreceptor of the D2-Type). n gives the number of analyzed cells; N gives the number of analyzed mice. Significances/p-values according to Chi-square test. P-values indicating significant differences are given in bold red.

	**NCS1-KO**			**NCS1-WT §**			
	**%**	**95% CI**	**n/N**	**%**	**95% CI**	**n/N**	**p-value**
**Desensitized D2-AR**	56.5	48.3-64.5	77/6	34.9	24.3-47.3	22/6	**0.006**
**Sensitized D2-AR**	43.5	35.5-51.8	61/6	65.1	52.9-75.7	41/6	
	**C57BL/6 DNIP**			**C57BL/6 srDNIP**			
	**%**	**95% CI**	**n/N**	**%**	**95% CI**	**n/N**	**p-value**
**Desensitized D2-AR**	64.8	51.5-76.2	37/5	32.3	23.8-42.2	31/6	**0.0002**
**Sensitized D2-AR**	35.2	23.8-48.5	17/5	67.7	57.8-76.2	65/6	

§: WT data were pooled as NCS-1+/+ littermate data were not significantly different to that from non-littermate NCS-1+/+, or from C57BL/6J mice.

Table S5: Comparison of pacemaker (frequency and precision), and dopamine responses of SN DA neurons in different glucose concentrations. Data from experiment shown in Figure 4, from C57BL/6 mice (PN13), in 2.5 mM or 25 mM glucose. Pacemaker frequencies are given as basal firing rate (mean firing rate during the first 10 minutes of recordings). Pacemaker precision is displayed as coefficient of variance of the interspike interval (CV-ISI). Mean relative activities in the last minute of dopamine are given as "activity at min 15 in DA [%]". "Pooled": all dopamine-inhibited neurons were analyzed as one population. "Desens": only SN DA neurons with desensitized inhibitory dopamine responses were analyzed. "Sens": only SN DA neurons with sensitized (sustained) inhibitory dopamine responses were analyzed. n gives the number of analyzed cells; N gives the number of analyzed mice. Data sets marked by (#) were not normally distributed. Significances/ p-values according to unpaired Mann-Whitney-test. P-values indicating significant differences are given in bold red.

	C57BL/6														
	2.5 mM glucose							25 mM glucose							
		**Mean**	**SEM**	**SD**	**Median**	**95% CI**	**n/N**		**Mean**	**SEM**	**SD**	**Median**	**95% CI**	**n/N**	**p-value**
**Basal firing rate [Hz]**	Pooled #	1.56	0.10	0.69	1.52	1.4-1.8	45/5	Pooled	1.85	0.08	0.59	1.88	1.7-2.0	58/4	**0.0155**
	Desens	1.73	0.18	0.78	1.65	1.4-2.1	20/5	Desens	1.90	0.10	0.65	1.86	1.7-2.1	38/4	0.2395
	Sens	1.42	0.12	0.58	1.41	1.2-1.7	25/5	Sens	1.74	0.11	0.47	1.90	1.5-1.9	20/4	**0.0366**
**CV-ISI [%]**	Pooled #	12.83	1.20	8.07	10.11	10.1-15.3	45/5	Pooled	11.14	0.85	6.45	10.90	9.4-12.9	58/4	0.4126
	Desens	12.27	1.68	7.50	9.98	8.8-15.8	20/5	Desens	11.38	1.10	6.79	10.36	9.1-13.6	38/4	0.6911
	Sens #	13.82	1.72	8.60	13.92	9.7-16.8	25/5	Sens	10.19	1.36	5.92	10.90	7.3-13.0	20/4	0.3005
**Activity at min 15 in DA [%]**	Pooled #	23.03	4.92	33.00	2.86	13.1-32	45/5	Pooled #	40.49	6.97	53.10	20.80	26.5-54.4	45/5	**0.0272**
	Desens	51.01	7.20	32.20	53.79	35.9-66.1	20/5	Desens #	61.47	8.92	55.00	58.48	43.4-79.6	20/5	0.5005
	Sens #	0.65	0.23	1.16	0	0.2-1.1	25/5	Sens #	0.63	0.27	1.22	0	0.06-1.2	25/5	0.7607

Table S6: Number and ratio of SN DA neurons with sensitized and desensitized inhibitory dopamine responses in 2.5 and 25 mM glucose. Data from experiment shown in Figure 4, and Table S5 (D2-AR dopamine autoreceptor of the D2-Type). n gives the number of analyzed cells; N gives the number of analyzed mice. Significances/p-values according to Chi-square test. P-value indicating significant differences is given in bold red.

	**Juvenile C57BL/6**					
	**2.5 mM glucose**			**25 mM glucose**		
	**%**	**95% CI**	**n/N**	**%**	**95% CI**	**n/N**
**Desensitized D2-AR**	44.4	30.9-58.8	20/5	66.7	53.7-77.5	38/4
**Sensitized D2-AR**	55.6	41.2-69.1	25/5	33.3	22.5-46.3	19/4
**p-value**	**0.0284**					

Table S7A/B: Comparison of pacemaker (frequency and precision), dopamine responses of SN DA neurons, and glucose-induced changes in pacemaker activity. Data from experiments shown in Figure 5A-C, derived from C57BL/6 mice (PN13). Glucose concentration in ACSF was transiently reduced from 25 mM to 1 mM and back to 25 mM. Pacemaker frequencies are given as basal firing rate (mean firing ate during the first 10 minutes of recordings). Pacemaker precision is displayed as coefficient of variance of the interspike interval (CV-ISI). Mean relative activities in the last minute of dopamine are given as "activity at min 15 in DA [%]". "All analyzed SN DA": all dopamine-inhibited neurons were analyzed as one population. "Sensitized SN DA": only SN DA neurons with sensitized (sustained) inhibitory dopamine responses were analyzed. "Desensitized SN DA": only SN DA neurons with desensitized inhibitory dopamine responses were analyzed. Table A gives the absolute mean values, Table B the mean absolute and relative changes. n gives the number of analyzed cells; N gives the number of analyzed mice. Data sets marked by (#) were not normally distributed. Significances/p-values according to paired Friedman test with Dunn´s multiple comparison post-hoc test. P-values indicating significant differences are given in bold red.

A

	**All analyzed SN DA**							
	**Glucose [mM]**	**Mean**	**SEM**	**SD**	**Median**	**95% CI**	**n/N**	**p-values**
**Basal firing rate [Hz]**	25	1.90	0.12	0.60	1.89	1.7-2.1	28/8	**<0.0001**
	1	1.46	0.11	0.55	1.41	1.2-1.7	28/8	**<0.0001**
	25	2.04	0.12	0.64	2.16	1.8-2.3	28/8	0.2306
**CV-ISI [%]**	25 #	9.36	1.24	6.34	7.72	6.8-11.9	28/8	**0.0005**
	1	13.01	1.33	6.76	11.25	10.3-15.7	28/8	**<0.0001**
	25	7.72	0.68	3.46	8.23	6.3-9.1	28/8	>0.9999
**Activity at min 15 in DA [%]**	25 #	53.51	8.25	43.68	54.98	36.6-70.4	28/8	Sens: 25%<br>Desens: 75%
	**Sensitized SN DA**							
	**Glucose [mM]**	**Mean**	**SEM**	**SD**	**Median**	**95% CI**	**n/N**	**p-values**
**Basal firing rate [Hz]**	25	1.55	0.17	0.44	1.51	1.1-1.9	7/8	>0.9999
	1	1.24	0.14	0.39	1.37	0.9- 1.6	7/8	**0.0458**
	25	1.73	0.19	0.51	1.86	1.3- 2.2	7/8	0.3264
**CV-ISI [%]**	25	13.73	3.00	7.94	13.58	6.4-21.1	7/8	>0.9999
	1	15.42	2.29	6.06	12.68	9.8-21.0	7/8	0.2364
	25	10.25	1.15	3.03	9.52	7.5-13.1	7/8	>0.9999
**Activity at min 15 in DA [%]**	25 #	0.11	0.08	0.22	0	-0.9-0.3	7/8	
	**Desensitized SN DA**							
	**Glucose [mM]**	**mean**	**SEM**	**SD**	**median**	**95% CI**	**n/N**	**p-values**
	25	2.02	0.14	0.61	2.09	1.7- 2.3	21/8	**<0.0001**
	1	1.53	0.13	0.58	1.50	1.3-1.8	21/8	**<0.0001**

**Basal firing rate [Hz]**	25	2.15	0.15	0.65	2.25	1.8-2.5	21/8	>0.9999
**CV-ISI [%]**	25 #	7.75	1.14	4.97	7.14	5.4-10.1	21/8	**0.0003**
	1	12.12	1.59	6.93	10.83	8.8-15.5	21/8	**<0.0001**
	25	6.80	0.73	3.19	6.64	5.3-8.3	21/8	>0.9999
**Activity at min 15 in DA [%]**	25	71.31	7.80	35.28	76.36	55.3-87.4	21/8	

B

**Change of …**	**All analyzed SN DA**							
	**Glucose [mM]**	**Mean**	**SEM**	**SD**	**median**	**95% CI**	**n/N**	**p-values**
firing rate [Hz]	25 to 1 #	0.44	0.08	0.40	0.31	0.3-0.6	28/8	**0.0491**
	1 to 25	0.58	0.09	0.44	0.50	0.4-0.8	28/8	
firing rate [%]	25 to 1 #	24.73	3.57	17.83	19.85	17.4-32-1	28/8	**<0.0001**
	1 to 25	51.66	9.83	49.16	38.88	31.4-72.0	28/8	
CV-ISI [%]	25 to 1	62.41	14.16	72.22	51.16	33.2-51.6	28/8	0.3533
	1 to 25	35.72	3.82	19.49	36.99	27.9-43.6	28/8	
	**Sensitized SN DA**							
	**Glucose [mM]**	**Mean**	**SEM**	**SD**	**median**	**95% CI**	**n/N**	**p-values**
firing rate [Hz]	25 to 1	0.31	0.12	0.32	0.33	0.01-0.6	7/8	0.3176
	1 to 25	0.49	0.11	0.30	0.52	0.2-0.8	7/8	
firing rate [%]	25 to 1	19.57	7.53	19.92	22.79	1.1-38.0	7/8	0.0728
	1 to 25	44.38	10.36	27.41	53.17	19.0-69.7	7/8	
CV-ISI [%]	25 to 1	49.82	41.39	109.5	49.57	51.5-151.1	7/8	0.7104
	1 to 25	29.56	7.11	18.11	32.95	12.2-47.0	7/8	
	**Desensitized SN DA**							
	**Glucose [mM]**	**Mean**	**SEM**	**SD**	**median**	**95% CI**	**n/N**	**p-values**
firing rate [Hz]	25 to 1 #	0.48	0.09	0.43	0.29	0.3-0.7	21/8	0.2648
	1 to 25	0.61	0.11	0.49	0.49	0.4-0.8	21/8	
firing rate [%]	25 to 1 #	23.46	4.27	19.08	18.35	14.5-32.4	21/8	**0.0326**
	1 to 25	51.81	12.14	54.28	31.37	26.4-77.2	21/8	
CV-ISI [%]	25 to 1	67.05	12.90	67.50	52.74	40.0-94.2	21/8	0.4699
	1 to 25	37.99	4.53	19.73	41.69	28.5-47.5	21/8	

Table S8: Dose-response curve fitting parameters (A), EC_{50} values (B), comparison of pacemaker frequency and dopamine responses (C) and glucose-induced changes (D) of SN DA neurons. Data from experiment shown in Figure 5D-G and S3, derived from C57BL/6 mice (PN13). "Sensitized SN DA": only SN DA neurons with sensitized (sustained) inhibitory dopamine responses were analyzed. "Desensitized SN DA": only SN DA neurons with desensitized inhibitory dopamine responses were analyzed. **(A)** Half maximal effective glucose concentrations (EC_{50} values), and Hill-slope, derived from fitting according to the Hill-equation (top and bottom were set to 1 and zero, respectively). The mean relative firing rates of all cells at each concentration were fitted ("Fit of means"), and also by the mean fit of the individual fits for each neuron ("Mean of individual fits"). **(B)** Gives mean EC_{50} values, derived from individual EC_{50} values of individual fits for each cell. **(C)** Pacemaker frequencies are given as basal firing rate (mean firing rate during the first 10 minutes of recordings). Mean relative activities in the last minute of dopamine are given as "activity at min 15 in DA [%]". **(D)** Change of pacemaker frequency is given in Hz and as percentage change between highest and lowest glucose concentrations. n gives the number of analyzed cells; N gives the number of analyzed mice. Data sets marked by (#) were not normally distributed. Significances/p-values according to paired Friedman-test or unpaired Kruskal-Wallis test with Dunn´s multiple comparison post-hoc test. P-values indicating significant differences are given in bold red.

A

		**EC_{50} value**			**Hill slope**			
		**mean**	**SE**	**95% CI**	**mean**	**SE**	**95% CI**	**n/N**
**All analyzed SN DA**	Fit of means	0.66	0.11	0.5-0.9	0.88	0.14	0.7-1.2	13/7
	Mean of individual fits	0.87	0.01	0.8-0.9	1.09	0.01	1.1-1.1	13/7
**EC_{50} < 1mM glucose**	Fit of means	0.35	0.04	0.3- 0.4	1.04	0.11	0.9-1.3	5/7
	Mean of individual fits	0.36	0.003	0.3-0.4	1.05	0.01	1.0-1.1	5/7
**EC_{50} > 1mM glucose**	Fit of means	2.16	0.23	1.7-2.7	1.44	0.22	1.1-1.9	8/7
	Mean of individual fits	2.23	0.016	2.2- 2.3	1.68	0.01	1.6- 1.7	8/7
**Sensitized SN DA**	Fit of means	0.35	0.05	0.2-0.5	1.06	0.16	0.8-1.5	3/7
	Mean of individual fits	0.34	0.003	0.3-0.4	1.05	0.01	1.0- 1.1	3/7
**Desensitized SN DA**	Fit of means	0.85	0.168	0.6-1.2	0.91	0.16	0.7- 1.3	10/7
	Mean of individual fits	1.04	0.013	1.0- 1.1	1.20	0.01	1.1- 1.2	10/7

B

	**Individual EC_{50} values**						
	**mean**	**SEM**	**SD**	**median**	**95% CI**	**n/N**	**p-values**
**All analyzed SN DA** #	1.06	0.29	1.04	0.51	0.43-1.7	13/7	All vs. sens: 0.826
**Sensitized SN DA**	0.36	0.08	0.14	0.31	0.02-0.7	3/7	All vs. desens: >0.999
**Desensitized SN DA**	1.27	0.35	1.11	0.95	0.5-2.1	10/7	Sens vs. desens: 0.503

C

	**All analyzed SN DA**							
	**Glucose [mM]**	**mean**	**SEM**	**SD**	**median**	**95% CI**	**n/N**	**p-values**
**Basal firing rate [Hz]**	25	1.78	0.23	0.70	1.95	1.2-2.3	13/7	0.1017
	0.5/1	1.4	0.18	0.54	1.61	0.9-1.8	13/7	**<0.0001**

	25	2.34	0.26	0.77	2.37	1.8-2.9	13/7	0.1017
**Activity at min 15 in DA [%]**	25 #	61.64	16.60	59.9	47.45	25.5-97.8	13/7	Sens: 33%<br>Desens: 77%
	**Sensitized SN DA**							
	**Glucose [mM]**	**mean**	**SEM**	**SD**	**median**	**95% CI**	**n/N**	**p-values**
**Basal firing rate [Hz]**	25	1.52	0.39	0.68	1.62	0.2-3.2	3/7	0.6620
	0.5/1	1.17	0.25	0.43	1.62	0.1-2.2	3/7	**0.0429**
	25	2.15	0.70	1.22	1.91	0.9-5.2	3/7	0.6620
**Activity at min 15 in DA [%]**	25	0	-	-	0	-	3/7	
	**Desensitized SN DA**							
	**Glucose [mM]**	**mean**	**SEM**	**SD**	**median**	**95% CI**	**n/N**	**p-values**
**Basal firing rate [Hz]**	25	1.90	0.30	0.73	2.06	1.1-2.7	10/7	0.2498
	0.5/1	1.51	0.24	0.59	1.66	0.9-2.1	10/7	**0.0016**
	25	2.43	0.23	0.56	2.55	1.8-3.0	10/7	0.2498
**Activity at min 15 in DA [%]**	25	85.41	15.72	49.7	86.83	49.8-121	10/7	

D

**Change of …**	**All analyzed SN DA**							
	**Glucose [mM]**	**Mean**	**SEM**	**SD**	**median**	**95% CI**	**n/N**	**p-values**
firing rate [Hz]	25 to 0.5/1 #	0.38	0.07	0.21	0.48	0.2-0.5	13	**0.0039**
	0.5/1 to 25	0.94	0.14	0.43	0.86	0.6-1.3	13	
firing rate [%]	25 to 0.5/1	19.59	3.15	9.45	22.51	12.3-26.9	13	**0.0039**
	0.5/1 to 25	75.04	13.39	40.18	68.10	44.2-105.9	13	
	**Sensitized SN DA**							
	**Glucose [mM]**	**Mean**	**SEM**	**SD**	**median**	**95% CI**	**n/N**	**p-values**
firing rate [Hz]	25 to 0.5/1	0.35	0.16	0.27	0.48	-0.3-1.0	3	0.25
	0.5/1 to 25	0.98	0.46	0.80	0.78	-1.0-3.0	3	
firing rate [%]	25 to 0.5/1	19.68	7.77	13.46	24.92	-13.8-53.1	3	0.25
	0.5/1 to 25	75.13	21.91	37.95	68.10	-19.2-169.4	3	
	**Desensitized SN DA**							
	**Glucose [mM]**	**Mean**	**SEM**	**SD**	**median**	**95% CI**	**n/N**	**p-values**
firing rate [Hz]	25 to 0.5/1	0.39	0.08	0.20	0.49	0.2-0.6	10	**0.0313**
	0.5/1 to 25 #	0.92	0.09	0.21	0.94	0.7-1.1	10	
firing rate [%]	25 to 0.5/1	19.54	3.42	8.38	19.84	10.7-28.3	10	**0.0313**
	0.5/1 to 25	74.99	18.29	44.80	64.64	28.0-122.0	10	
